# Understanding and Using the Brief Implicit Association Test: Recommended Scoring Procedures

**DOI:** 10.1371/journal.pone.0110938

**Published:** 2014-12-08

**Authors:** Brian A. Nosek, Yoav Bar-Anan, N. Sriram, Jordan Axt, Anthony G. Greenwald

**Affiliations:** 1 University of Virginia, Charlottesville, VA, United States of America; 2 Center for Open Science, Charlottesville, VA, United States of America; 3 Ben-Gurion University of the Negev, Beer-Sheva, Israel; 4 Implisci, Charlottesville, VA, United States of America; 5 University of Washington, Seattle, WA, United States of America; The University of New South Wales, Australia

## Abstract

A brief version of the Implicit Association Test (BIAT) has been introduced. The present research identified analytical best practices for overall psychometric performance of the BIAT. In 7 studies and multiple replications, we investigated analytic practices with several evaluation criteria: sensitivity to detecting known effects and group differences, internal consistency, relations with implicit measures of the same topic, relations with explicit measures of the same topic and other criterion variables, and resistance to an extraneous influence of average response time. The data transformation algorithms *D* outperformed other approaches. This replicates and extends the strong prior performance of *D* compared to conventional analytic techniques. We conclude with recommended analytic practices for standard use of the BIAT.

## Understanding and Using the Brief Implicit Association Test: I. Recommended Scoring Procedures

Even the most brilliant research ideas can flounder if data collection procedures and data analytic strategies applied in the pursuit of these ideas are suboptimal. Research efficiency, the knowledge gained in proportion to resources invested, can be improved by maximizing the quality of procedural and analytical methods. Numerous paradigms in mental chronometry, such as Stroop and lexical-decision tasks, define constructs derived from contrasting response latencies between performance conditions [1]. The Implicit Association Test (IAT) [2] is a chronometric procedure that quantifies strength of conceptual associations by contrasting latencies across conditions [3]. Participants categorize stimuli representing four categories (e.g., Democrats, Republicans, good words, bad words) in two different conditions – (a) categorizing Democrats and good words together with one response key, and Republicans and bad words together with another response key; and (b), categorizing Republicans and good words together with one response key, and Democrats and bad words with the other. The difference in average response latency between conditions is taken as an indicator of differential association strengths among the concepts. Since its introduction, the IAT has gained in acceptance and influence, and implicit measures generally have had a wide impact on behavioral research [4]–[6].

Even when the procedures in a given mental chronometric paradigm are defined unambiguously, there may be various methods to derive scores quantifying the construct of interest. Different scoring practices may lead to unique findings, and across articles it may be difficult to identify scoring procedures as responsible for producing distinct effects. Also, in the absence of standard analytic procedures, researchers may drift into exploratory search and inflate false positives by converging on that scoring strategy that reveals an effect most consistent with the hypothesis. Therefore, standards regarding the scoring procedures contribute to the integrity of research. Ideally, a standard method maximizes reliability and validity of the resulting scores and findings.

Originally, the IAT score, like many other chronometric constructs, was defined as the mean latency (log latency) difference between conditions. Subsequently, candidate scoring procedures for the IAT have been evaluated on numerous criteria [7]. Compared to the original IAT score, the recommended IAT *D* score improved the sensitivity and power of the measure (e.g., a 38% decrease in needed sample size to detect the average correlation) [7]. The present article similarly evaluates candidate scoring procedures for the Brief Implicit Association Test (BIAT) [8]. The goal of this investigation is to determine optimal data analytic strategies for deriving association scores from the BIAT.

### The Brief Implicit Association Test

The BIAT was developed to shorten the time required to measure associations, while retaining some of the valuable design properties of the IAT. The BIAT can use as few as two response blocks of 20 trials each, which can be completed in a little over a minute. The design that we evaluate here is a sequence of four response blocks of 20 trials each that is preceded by a 16-trial warm-up block (see Table 1).

**Table 1:** BIAT procedure.

Block	Trials	Trial structure	Example focal	Example non-focal
1	16	4 attribute only + 12 trials alternating category and attribute	Good words (attribute) and mammals (category)	Bad words (attribute) and birds (category)
2	20	4 attribute only + 16 trials alternating category and attribute	Good words (attribute) and Democrats (category)	Bad words (attribute) and Republicans (category)
3	20	4 attribute only + 16 trials alternating category and attribute	Good words (attribute) and Republicans (category)	Bad words (attribute) and Democrats (category)
4	20	4 attribute only + 16 trials alternating category and attribute	Good words (attribute) and Democrats (category)	Bad words (attribute) and Republicans (category)
5	20	4 attribute only + 16 trials alternating category and attribute	Good words (attribute) and Republicans (category)	Bad words (attribute) and Democrats (category)

Notes: Procedure displays for a good-focal political attitude measure. The order of blocks 2 and 4 with blocks 3 and 5 was counterbalanced. A trial begins with the onset of the stimulus and ends once a correct categorization is made. Clarity between the two dimensions (Democrats/Republicans and Good/Bad) was enhanced by presenting the labels and stimulus items from each dimension in a different color or stimulus format (e.g., Democrats/Republicans as images; Good/Bad as words).

**Figure 1:**
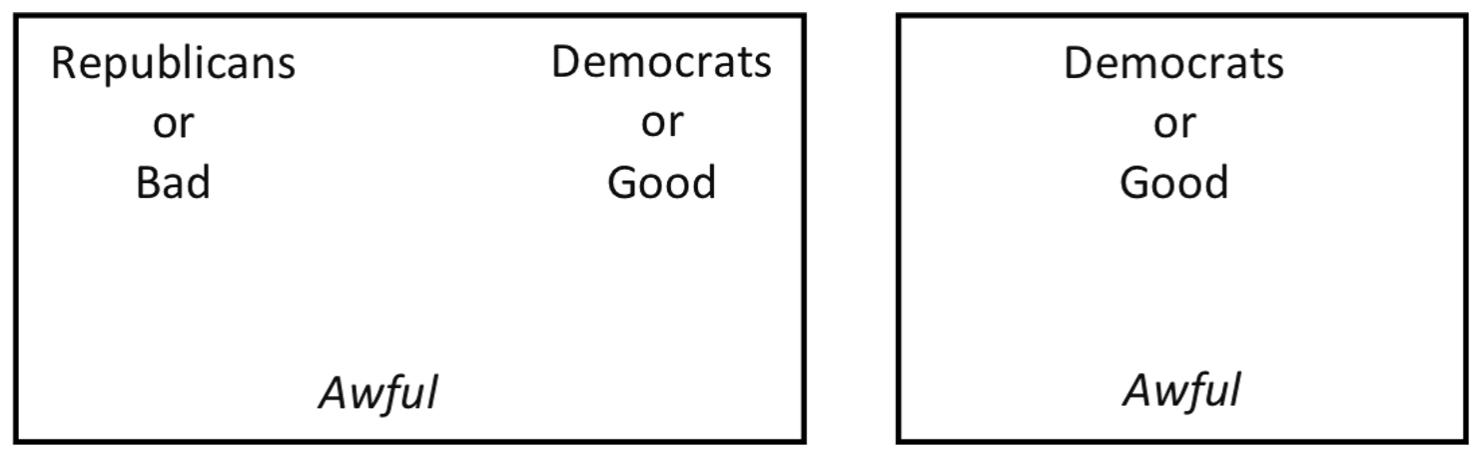
Schematics of the same single response trial of one block of the IAT on the left, and the BIAT on the right. In the IAT, the correct response is the left key because *Awful* belongs to the Category Bad. In the BIAT, the correct response is the left key because *Awful* does not belong to the categories Democrats or Good.

The categories and exemplars used in the BIAT and the mapping of category exemplars to response keys are the same as those used in the combined blocks of the IAT (see [9] for a full description of the standard IAT procedure). Both procedures use items from four categories (e.g., Democrats, Republicans, good words, bad words) and, within a block, each item is mapped to one of two responses. Whereas in the IAT each category is explicitly associated with one of the two response options, in the BIAT, participants focus on just two of the four categories. Items from these two *focal* categories are categorized with one response key (e.g., the “i” key), and any other items that appear on the screen (*non-focal*) are categorized with the other response key (e.g., the “e” key; see Fig. 1). In the two blocks, the focal attribute is kept fixed (e.g., using either *Good* or *Bad* in the case of attitude) and the two contrasted concepts (Democrats, Republicans) are focal in separate blocks. These design changes simplify instructions and decrease the need for practice shortening total administration time.

The ability of the BIAT to function effectively as a measure of attitudes, identities, and stereotypes has been previously established [1]. Also, the BIAT has found application in many studies already [10]–[13]

The present studies analyzed data from a very large data collection in a study that administrated a random selection of measures of attitudes regarding race, politics and the self from a large pool of possible indirect and direct attitude measures [14], comparing the psychometric qualities of seven different indirect measures on a wide variety of criteria. The BIAT was the best of the seven measures in 8 out of the 29 criteria used to evaluate the measures as measures of attitudinal preference, and the second best in another 8 criteria (always outperformed only by the IAT). The BIAT had the best average ranking (2.34), slightly better than the IAT (2.39). These results suggest that the BIAT is a highly useful measure for research application. Establishing the best practices of analyzing BIAT data is essential for optimizing the usefulness of the BIAT.

### Evaluation Criteria

#### Sensitivity to known effects–main effects and group differences

All else being equal, better scoring procedures should be more sensitive to the measured construct. Comparing among scoring methods of the same data, eliciting a larger overall effect magnitude was considered a desirable criterion. Two of the three topics for the present study – racial attitudes and self-esteem – were appropriate for this criterion. Both elicit strong effects favoring whites to blacks and self to others respectively [15]–[17].

There are known group differences in political and racial attitudes. The true score of the group difference will be underestimated by error in measurement and analysis, except for the unlikely scenario that the measurement error is confounded with group membership (see [7] for a more in-depth discussion of this point). Reducing error in analysis will therefore maximize the magnitude of the group difference estimate by moving it closer to the true score. The third topic – political attitudes – is polarized with liberals or Democrats preferring Democrats and conservatives or Republicans preferring Republicans, even implicitly [18]–[19]. Better scoring algorithms will be more sensitive to detecting that group difference. Likewise, Black and White people have different implicit racial attitudes – each more inclined to show positivity toward their own racial group, though Whites more so – providing another group difference criterion [16]. For implicit self-esteem consistent group differences are not observed in the reported literature. For example, while self-reported self-esteem differs between people from Eastern and Western cultures, this difference is not observed implicitly [17].

#### Internal consistency

Better scoring procedures should maximize the internal consistency of the measurement. The BIAT had four response blocks, two for each condition. Independent scores were computed for the first pair and second pair of response blocks. The correlation of these scores was the measure of internal consistency. Scoring algorithms that elicited higher internal consistencies with the same data were favored over those that elicited lower internal consistencies.

#### Relations with other implicit measures of the same topic (convergent validity)

The data of the present studies offered an opportunity to examine the correlation of the BIAT with seven other procedures for implicit measurement. Stronger correlations with other implicit measures of the same topic indicated better performance by the BIAT scoring procedures.

#### Relations with parallel self-report measures and criterion variables

Better scoring procedures will elicit stronger relations with known correlates of a measure than worse scoring procedures. For example, height and weight are distinct, correlated constructs. Assessments that minimize random error in measurement of height and weight will maximize their correlation, getting it closest to the true correlation (assuming that the assessments are not influenced by the same extraneous influence; see also [7]). The data collection included self-reported attitudes and other direct measurements of known covariates for each of the topics.

Dual-process and dual-system perspectives in attitudes research presume that implicit and explicit attitudes are distinct constructs – the former measured indirectly with procedures like the BIAT, the latter measured directly with self-report procedures (see [5]-[6] for a review). Justification of distinct implicit and explicit constructs requires evidence of divergent validity. Nevertheless, it is well established that these constructs are related [20]. The ceiling for maximizing the relationship is the true correlation. In most cases, this correlation will be less than 1.0 because implicit and explicit measures do not assess the same construct [21]. Thus, like height and weight, the best measure of both will maximize their relationship by minimizing random error. Separate evidence is required to justify the interpretation of the measures as assessments of distinct constructs (for more in-depth treatment of this topic see ([21], [22]).

#### Resistance to extraneous influences

Extraneous influences are procedural or other factors that produce variation in measurement that is unrelated to the construct of interest. Two extraneous influences are common for response latency measures: participants' average response time, and the order of the measurement blocks. Participants who respond more slowly on average also tend to show larger effects on many response latency measures, especially when computing difference scores [23]. Also, the order of measurement blocks is a well-known influence on response latency measures like the IAT [2], [24]. It is more desirable to have a scoring procedure that is less sensitive to these influences. Ultimately, in the present studies, the order effect did not serve as a criterion variable because the procedural design effectively eliminated that common extraneous influence (see S1 File), so we examined only the average speed of responding.

### Candidate Data Transformations

Four candidate data transformations were compared: mean difference of average latencies, mean difference of average reciprocals, mean difference of log transformed latencies, and *D*. A fifth data common data transformation, differences between median latencies, was not tested because it performed so poorly in prior comparative analyses [7].

#### Difference scores of mean untransformed or transformed latencies

The most straightforward method for comparing average response latency between contrasted conditions is to average the response latencies in each condition and subtract one from the other. Many research applications apply a data transformation log or reciprocal (inverse) to the raw latencies prior to averaging. Whether transformed or untransformed, these approaches are vulnerable to intra- and inter-individual biases on difference scores [23]. As such, we expected that these would not be among the best performing algorithms.

#### *D* algorithm

Greenwald and colleagues [7] introduced the *D*-algorithm as a substantial improvement for scoring the IAT. For the BIAT, the *D* computation is the same. *D* is the difference between the average response latencies between contrasted conditions divided by the standard deviation of response latencies across the conditions (distinct from the pooled within-conditions standard deviation). Functionally, it is an individual effect size assessment that is similar to Cohen's *d* except, with the same number of trials per condition, *D* has a theoretical minimum of −2 and maximum of +2 when blocks of the same size are compared [3]. See Table 2 for instructions on how to calculate a BIAT *D* score. With the IAT, *D* reduces the impact of extraneous influences like average response latency [25]–[26], and increases sensitivity to detecting relations with known covariates [7]. In addition to the *D* calculations, we examined a variety of data treatments such as exclusion decisions and error trial handling.

**Table 2:** A step-by-step guide for calculating the recommended D score from Table 8.

	n_1_ latencies from condition 1 are contrasted with n_2_ latencies in condition 2, n_1_ + n_2_ = N
	Steps for calculating D
1	Compute the standard deviation of the N latencies, SD.
2	M_1_ is the mean of the latencies in condition 1. M_2_ is the mean of the latencies in condition 2.
3	*D = (M_2_−M_1_)/SD*

### Other Data Treatment Considerations

The data transformation is only one of a variety of possible data treatment decisions for analysis. We evaluated four additional criteria in the order presented below.

#### Warm-up trials

Sriram and Greenwald [8] defined the four initial trials of each response block that only presented target concepts as practice trials to learn the performance rules for that block. They deleted these trials prior to calculation of BIAT scores. For short response blocks, this is a relatively large amount of data loss. In Study 1, we evaluate whether the warm-up trials provide added value for construct measurement.

#### Errors

When a participant presses the wrong key in response to a stimulus item, the task presents a red X and waits for the correct response to be made. In the IAT, Greenwald and colleagues [2], [7] found that error responses contain useful information for measuring the intended construct. For blocks with more difficult response configurations, participants are likely to go slower *and* make more errors than blocks with easier response configurations. As such, incorporating errant responses that are delayed by the need to correct them may have positive benefits for measuring association strengths with the BIAT. In Study 5, we compared the effects of deleting error trials or retaining them “as is” (recording the response latency from the beginning of the trial to the correct response regardless of whether an error was made).

#### Very fast and very slow response trials

Extreme responses – either very slow or very fast – can indicate inattention to the task performance rules. It is not possible, for example, for humans to process and respond to stimulus items with the BIAT rules faster than about 200 ms. Likewise, taking more than 10,000 ms to make a response is unlikely to occur when the participant is attending to the task. In Study 3, we tested cut-offs for very slow and very fast response latencies, and compared treatments of deleting versus recoding the outliers to the cut-off boundary response latency.

#### Exclusion criteria for overall task performance

Separate from computing an individual score, researchers typically consider a variety of criteria for excluding all of the data from a given task or participant. For example, if a participant is sufficiently disinterested or unable to adhere to the task instructions his or her performance data may be sufficiently invalid so that its inclusion in analyses impairs criteria of efficiency and validity. For example, some participants may press the keys quickly at random to get through the task as rapidly as possible, paying no attention to accuracy. Identifying and removing such non-cooperative participants can improve the validity of a data set. At the same time, unless there is compelling reason to do so, it is good practice to retain as many participants as possible. Study 4 tested different exclusion rules for error rates, number of very fast response latencies, and number of very slow response latencies.

### Data Source

Participants were volunteers from the Project Implicit participant pool (https://implicit.harvard.edu/; see [20] for more information). Participants register an identity and are randomly assigned to a study from the pool each time they visit the site. The participant sample is very diverse, but not representative of any identifiable population.

The present data came from a very large data collection termed “Attitudes 3.0” [14] collected for a defined period of time to gather a large sample from November 6, 2007 to May 30, 2008. In Attitudes 3.0, each study session was comprised of a random selection of measures from a large pool of possible measures. The total session time was designed to be approximately 15 minutes. Each session administered a small subsample of the available measures. The measures assessed evaluations related to three topics: political attitudes, racial attitudes, and self-esteem (see [14] for a full description of the procedure, measures, and constraints on the random selection process). Participants could complete the study multiple times. Each time, the participant would receive a new random selection of measures. In total, there were almost 40,000 sessions. For the present studies, we selected the sessions in which participants completed at least one of the three Brief IATs – political attitudes, racial attitudes, and self-esteem. For each topic, there were more than 2,000 completed BIATs.

### Overview of Studies

We conducted 7 studies and multiple replications to evaluate data treatment alternatives for the BIAT. This provides an opportunity to enhance the psychomentric properties of the BIAT, and to replicate and extend the observation that the *D* scoring algorithm [7] outperformed a variety of conventional analytic techniques with a variant of the IAT. Here, we provide a full report of the studies using the politics data and briefly summarize replication studies with race and self-esteem as target concepts (materials and data are available at http://osf.io/7A2n8/).

Studies 1 and 2 examined psychometric properties of four data transformations, which are described below. Study 1 demonstrated that retention of four warm-up trials for each response block at least slightly damaged psychometric criteria, on the basis of which these warm-up trials were removed from the data for all remaining studies. Study 2 compared the four transformations in terms of possible contamination by subjects' overall speed of responding, finding that one was clearly superior to the other three, the *D* algorithm. As a consequence, the remaining studies focused on the superior D algorithm. Study 3 considered alternatives for dealing with extreme latency trials – very fast or very slow, finding that the D algorithm was only mildly affected by latency tail treatments, but also that the D algorithm could be slightly improved by reducing the impact of fast or slow outliers. Study 4 found that including those with more than 10% of trials being very fast (<300 ms) disrupted psychometric properties of the BIAT enough to warrant excluding them. High error rates (>30%) also reduced sensitivity but not substantially. On the basis of Study 4, results for all studies are reported excluding respondents who had more than 10% of trials faster than 300 ms. Study 5 established that retaining error trials was superior to removing them. Study 6 found that the “good-focal” response blocks were considerably more reliable and valid than “bad-focal” response blocks, confirming the result previously reported by Sriram and Greenwald [8]. As a consequence of that finding, results from Studies 1–5 are reported in text for BIATs that used good-focal response blocks only. S1-S4 Tables report additional findings with the bad-focal blocks. These were highly consistent with the good-focal BIAT results. Study 7 showed that data from the first and second of two replications of the BIAT procedure were comparable in psychometric properties. The General Discussion summarizes the recommended analytic practices based on this investigation.

## Method for Political Attitude Studies

### Participants

2,358 study sessions of the Attitudes 3.0 dataset included the politics Brief IAT with at least 4 completed blocks for either good-focal or bad-focal tasks. Average age of the participants was 29.4 (SD  =  12.1), 65% were female, and 73.1% were White, 6.5% Black, 3.1% east Asian, 2.2% south Asian, 6.7% multiracial, and the remainder other or unknown.

### Measures

#### BIAT

In the Brief IAT, two categories (e.g., *Democrats* and *good words*) are "focal". Stimulus items appear one at a time in the middle of the screen and participants must categorize the stimulus items as either belonging to one of the focal categories (press the ‘i’ key) or not (press the ‘e’ key). If the participant makes an error, a red "X" appears below the stimulus and the trial continues until the correct key is pressed. In this study, the stimulus items that appeared but did not belong to the focal categories were always the contrasting stimuli for the other tasks (e.g., *Republicans* and *bad words* when *Democrats* and *good words* were the focal categories).

To evaluate both good and bad-focal conditions, the Brief IAT sequence included nine blocks of trials (Table 1). In each block, the first four trials were selected from the target categories (e.g., *Democrats, Republicans*). The remaining trials for each block alternated between target categories and attributes (good, bad words). The first block was a practice round of 16 total trials with *mammals* and *good words* as the focal categories and *birds* and *bad words* as non-focal categories. The other eight blocks had the four category-only warm-up trials, and then 16 category-attribute alternating trials. The 2nd through 5th blocks had the same focal attribute (e.g., *good words*) and alternated the focal category (*Democrats, Republicans*) such that one appeared in blocks 2 and 4, and the other appeared in blocks 3 and 5. The 6th through 9th blocks had the other attribute focal (*bad words*) and likewise alternated the focal category between blocks. The order of attributes and categories as focal was randomized between subjects resulting in four between-subjects conditions (*good* or *bad* first; *Democrats* or *Republicans* first) for each topic (politics, race, self-esteem).

Sriram and Greenwald [8] observed that the Brief IAT showed stronger construct validity for response blocks when *good* was focal compared to when *bad* was focal. This experimental design enabled comparison of good and bad blocks for replication of this effect with a variety of evaluation criteria. Study 6 strongly confirmed Sriram and Greenwald's observation that good-focal blocks outperformed bad-focal blocks.

#### Other implicit measures

In addition to Brief IATs measuring the three topics of interest, participants were randomly assigned to complete one or more of seven other implicit measures about the same topics – the IAT, Go/No-go Association Task (GNAT) [27], single-target (ST-IAT) [28], Sorting Paired Features [29], Evaluative Priming (EPT) [30], Affect Misattribution Procedure (AMP) [31] and – a direct measure with time pressure – speeded self-report (SPD) [32]. A full report of the procedural details of each implicit measure appears in Bar-Anan and Nosek [14].

#### Self-reported attitudes and individual difference measures

Each participant received a random selection of self-report measures including (a) 7-point relative preference for Democrats compared to Republicans, White people compared to Black people, and Self compared to Other; (b) 11-point warmth ratings for each of those target concepts independently; (c) liking rating of 5 exemplars of Democrats, Republicans, Black people or White people, averaged within topic for analysis (Range  =  0 to 8); (d) 14-item Right-Wing Authoritarianism scale [33] (Range  =  1 to 6); (e) Modern Racism Scale [34] (Range  =  1 to 6); (f) Rosenberg Self-Esteem [35] (Range  =  1 to 6); (g) self-attributes questionnaire [36] (Range  =  1 to 7); (h) reported 2004 U.S. presidential vote (Kerry or Bush) and 2008 U.S. president voting intention (Democratic or Republican); (i) reported frequency of friendly contact with black people (Range  =  1 to 6); and (j) reported recency of receiving positive and negative feedback from others in daily life (Range  =  1 to 6; see Bar-Anan & Nosek [14] for comprehensive detail on the measures). All variables were coded so that positive correlations would indicate a relationship with the BIAT in the predicted direction. In the original study design, recency of positive feedback was predicted to have a positive relation to implicit self-esteem – more recent explicitly reported positive feedback predicting higher implicit self-esteem. The empirical result was a weak relationship in the opposite direction. For the present analyses, we followed the empirical result for evaluation of candidate algorithms.

#### Demographics

During registration, participants reported a variety of demographic characteristics. Two of those were relevant for the present study: race (categorical identification including Black/African American and White/Caucasian), and political ideology (7-point scale from strongly conservative to strongly liberal).

### Procedure

Participants registered for the research participant pool at Project Implicit and completed a demographics questionnaire. On each subsequent visit to the site, participants were randomly assigned to studies from those presently available in the pool. Participants randomly assigned to this study were given a random selection of implicit and self-report measures that required a total time of about 15 minutes to complete.

### Data Preparation

Study sessions with at least one completed BIAT were retained. Response trials greater than 10,000 milliseconds indicate inattention to the task and were removed (456 of the total of 379,800 trials were removed, with removals disproportionately from the block-warm-up trials, which were 20% of trials and 48% of removals).

### Ethics Statement

The studies were approved by the University of Virginia Institutional Review Board to protect human participants.

## Study 1: Including vs. Removing Warm-up Trials

Sriram and Greenwald [8] removed the first four trials of each BIAT response block presuming that the shortened overall format of the procedure would make those trials particularly unreliable and vulnerable to irrelevant influences. We removed the first four trials in each block because there are no attribute words (e.g., Good or Bad) presented. Since such warm-up trials are qualitatively different from all other trials in a block, we investigated the effects of removing or retaining these trials.

However, with each block being just 20 total trials, removal of the first 4 trials is a substantial 20% reduction of the available data. Study 1 tested whether the initial trials could contribute to the measure's validity by comparing the performance of the BIAT with and without the first four trials. Like most other analyses presented in this article, it used data from which trials with latencies greater than 10,000 ms had been filtered and excluded subjects whose non-cooperation with instructions was indicated by their having more than 10% of latencies faster than 300 ms.

### Results and Discussion

Analyses summarized in text are only for good-focal blocks. Similar findings were observed for analyses of data from bad-focal blocks (Table 3). The findings showed that the warm-up trials provided no useful data. Across the four candidate data transformations, removing the first four trials left sensitivity in the BIAT to differences across political ideology unchanged (average *r*s  = .524 and .527 for warm-up trials retained and discarded respectively). Also, the internal consistency of the BIAT was slightly higher without the first four trials (average αs  = .753 and .743). BIAT correlations with other implicit measures were unaffected by removing the first four trials (average *r*s  = .554 and .563). Finally, BIAT correlations with parallel self-report measures and criterion variables were not different with and without the first four trials (average *r*s  = .552 and .558). Similar results were obtained with racial attitude measures (S1 Table), and with self-esteem measures (S2 Table).

**Table 3:** Comparison of retaining or removing 1 st four trials of each block (Study 1), candidate data transformations (Study 2), and bad versus good focal blocks (Study 6) across evaluation criteria for political attitudes.

		Retain all trials				Remove 1st four trials of each block				Average across algorithms	
	N	D	Reciprocal Diff	Log Diff	Latency Diff	D	Reciprocal Diff	Log Diff	Latency Diff	All Trials	Remove 1st four
**KNOWN GROUP DIFFERENCES**											
Political Ideology (Bad Focal)	2026	0.317	0.353	0.316	0.226	0.378	0.393	0.365	0.282	0.304	0.355
Political Ideology (Good Focal)	2026	0.548	0.557	0.533	0.453	0.557	0.556	0.535	0.457	0.524	0.527
**INTERNAL CONSISTENCY**											
Alpha (Bad Focal)	2012	0.506	0.612	0.550	0.399	0.564	0.623	0.557	0.389	0.521	0.538
Alpha (Good Focal)	2024	0.750	0.779	0.762	0.672	0.773	0.778	0.763	0.690	0.743	0.753
**RELATIONS WITH OTHER IMPLICIT MEASURES**											
*BAD FOCAL*											
IAT	290	0.392	0.454	0.392	0.262	0.472	0.488	0.439	0.315	0.377	0.431
GNAT	251	0.484	0.521	0.484	0.354	0.556	0.558	0.533	0.419	0.463	0.519
ST-IAT	244	0.357	0.391	0.350	0.244	0.400	0.404	0.371	0.281	0.337	0.365
SPF	271	0.420	0.463	0.413	0.314	0.471	0.487	0.447	0.354	0.404	0.441
EPT	262	0.304	0.342	0.282	0.179	0.350	0.371	0.318	0.229	0.278	0.318
AMP	361	0.227	0.248	0.195	0.118	0.272	0.276	0.228	0.151	0.197	0.232
SPD	417	0.321	0.361	0.326	0.233	0.375	0.381	0.359	0.289	0.311	0.352
											
*GOOD FOCAL*											
IAT	290	0.610	0.619	0.586	0.477	0.645	0.629	0.602	0.505	0.576	0.598
GNAT	251	0.648	0.664	0.639	0.553	0.676	0.668	0.652	0.578	0.628	0.645
ST-IAT	244	0.549	0.595	0.558	0.423	0.607	0.591	0.561	0.419	0.534	0.548
SPF	271	0.627	0.612	0.588	0.487	0.619	0.604	0.584	0.496	0.581	0.578
EPT	262	0.535	0.549	0.506	0.422	0.561	0.555	0.516	0.427	0.505	0.517
AMP	361	0.481	0.505	0.475	0.393	0.484	0.497	0.463	0.365	0.464	0.454
SPD	417	0.588	0.609	0.587	0.511	0.617	0.601	0.588	0.527	0.575	0.584
											
Bad focal average		0.360	0.400	0.352	0.245	0.418	0.428	0.389	0.293	0.341	0.383
Good focal average		0.579	0.595	0.565	0.468	0.604	0.594	0.569	0.477	0.554	0.563
**RELATIONS WITH SELF-REPORT MEASURES AND CRITERION VARIABLES**											
*BAD FOCAL*											
Dem-Rep Preference	396	0.358	0.390	0.362	0.264	0.430	0.427	0.410	0.316	0.344	0.397
Warmth for Democrats	401	0.277	0.320	0.315	0.256	0.341	0.343	0.345	0.289	0.292	0.330
Warmth for Republicans	398	0.339	0.363	0.324	0.223	0.401	0.394	0.375	0.293	0.313	0.366
Right-Wing Authoritarianism	438	0.411	0.455	0.411	0.281	0.451	0.480	0.456	0.364	0.391	0.439
Avg liking of 5 Democrats	335	0.238	0.325	0.253	0.137	0.327	0.369	0.318	0.219	0.239	0.309
Avg liking of 5 Republicans	335	0.300	0.363	0.323	0.233	0.360	0.397	0.374	0.305	0.305	0.359
Intended Vote in 2008 (D or R cand.)	375	0.265	0.291	0.261	0.177	0.321	0.324	0.303	0.221	0.249	0.293
Vote in 2004 (Bush or Kerry)	216	0.364	0.400	0.359	0.247	0.450	0.444	0.423	0.323	0.344	0.411
											
*GOOD FOCAL*											
Dem-Rep Preference	396	0.659	0.669	0.642	0.544	0.672	0.667	0.646	0.556	0.631	0.637
Warmth for Democrats	401	0.441	0.463	0.451	0.390	0.452	0.456	0.449	0.392	0.437	0.438
Warmth for Republicans	398	0.554	0.574	0.550	0.464	0.572	0.575	0.558	0.480	0.537	0.547
Right-Wing Authoritarianism	438	0.528	0.530	0.488	0.397	0.538	0.528	0.495	0.408	0.487	0.494
Avg liking of 5 Democrats	335	0.556	0.565	0.558	0.497	0.567	0.561	0.554	0.491	0.545	0.544
Avg liking of 5 Republicans	335	0.596	0.622	0.593	0.491	0.617	0.628	0.598	0.491	0.578	0.586
Intended Vote in 2008 (D or R cand.)	375	0.547	0.548	0.547	0.490	0.562	0.550	0.548	0.491	0.533	0.538
Vote in 2004 (Bush or Kerry)	216	0.663	0.654	0.651	0.594	0.692	0.659	0.657	0.597	0.641	0.653
											
Bad focal average		0.320	0.364	0.327	0.228	0.386	0.398	0.377	0.292	0.311	0.364
Good focal average		0.572	0.582	0.563	0.486	0.589	0.582	0.567	0.491	0.552	0.558
**RELATIONS WITH EXTRANEOUS INFLUENCE**											
*BAD FOCAL*										Average of absolute values	
Relation with average reciprocal	2055	0.053	0.151	−0.090	−0.336	0.089	0.172	−0.065	-0.324	−0.059	−0.035
Relation with average log	2055	−0.064	−0.166	0.115	0.421	−0.121	−0.201	0.076	0.401	0.083	0.044
Relation with average latency	2055	−0.062	−0.144	0.132	0.455	−0.115	−0.180	0.090	0.437	0.104	0.065
											
*GOOD FOCAL*										Average of absolute values	
Relation with average reciprocal	2063	0.080	0.110	−0.111	−0.318	0.067	0.105	−0.114	−0.331	−0.062	−0.071
Relation with average log	2063	−0.116	−0.132	0.124	0.384	−0.100	−0.137	0.121	0.396	0.070	0.075
Relation with average latency	2063	−0.115	−0.109	0.137	0.406	−0.095	−0.118	0.129	0.415	0.086	0.089

Correlations averaged after Fisher's z-transformation and then converted back.

Performance on the several evaluation criteria varied substantially across the four candidate transformations. For example, the correlation with political ideology with warm-up trials removed ranged from .457 (latency) to .557 (*D*). Internal consistency ranged from .690 (latency) to .778 (reciprocal). Average correlations with other implicit measures ranged from .477 (latency) to .604 (*D*). And average correlations with parallel self-report measures ranged from .491 (reciprocal) to .589 (*D*). In general, *D* was superior in these psychometric criteria to the other three measures. Logarithm was consistently close to these and reciprocal generally last. The poor performance of the reciprocal measure was almost certainly due to the weight it accords to fast responses as it performs closer to the others when latencies <400 milliseconds are removed (see Table 3).

The greatest effect of removing the four warm-up trials on any of the psychometric criteria was a slight increase in internal consistency, indicating that the initial four trials of each response block did not contribute positively to reliability and validity. Removing them is therefore a sensible analytic practice. Data analyses for subsequent studies reported here therefore also removed the four warm-up trials.

## Study 2: Sensitivity to Average Speed of Responding

Evaluations of the sensitivity of the four potential data transformation procedures to respondent differences in average latency of responding used the method of constructing *latency operating characteristics* similar to those reported in Figures 1 and 2 of Greenwald et al. [7]. For this purpose each subject's average latency in milliseconds was used, excluding from that computation the latencies for the four warm-up trials and latencies slower than 10s. Very similar findings are obtained when overall average reciprocal or overall average log latency are used as the indicator of average speed of responding.

**Figure 2:**
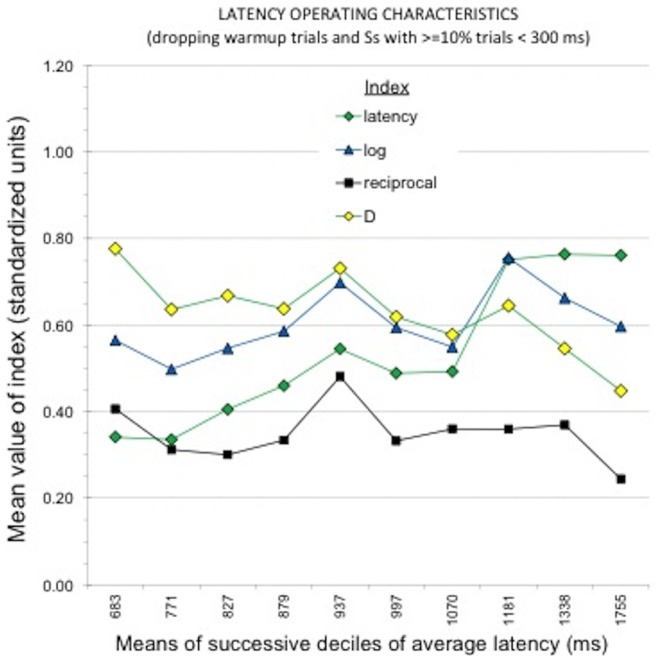
Latency operating characteristics, showing variation in mean standardized values of the five candidate BIAT scoring algorithms across deciles of the sample's distribution of average speed of responding for the political BIAT. For this plot, the algorithms were computed after deleting 4 warm-up trials from each response block and also deleting latencies greater than 10,000 ms. There were 202 or 203 respondents in each decile. Most noticeable in the graph is the inferior performance (smaller effect sizes) for the reciprocal measure, and strongest performance for the *D* measure. Also noticeable is that the D measure was smallest for the slowest subjects, whereas the log and latency measures were largest for the slowest subjects.

### Results and Discussion

On the basis of Study 1, we removed data for the first four trials of each response block, in addition to latencies slower than 10,000 ms and excluding subjects with more than 10% of responses faster than 300 ms.

#### Sensitivity to sample central tendency

The population sampled for this research was known to be politically liberal. On a scale ranging from –3 (strongly conservative) to 3 (strongly liberal), the sample mean was 0.93 (*N*  =  2,232, *SD*  =  1.64; for difference from 0, *t_2231_*  =  25.76, *p*  =  10^–137^). It was therefore expected that means for the political BIAT should be numerically positive, reflecting the ideologically liberal preference in the sample. Fig. 2 presents a latency operating characteristic for mean values of each transformation, simultaneously displaying differences among the transformations in magnitudes of effect sizes for the mean (higher is better) and in stability of the mean across variations in subjects' overall speed of responding. To enable comparison among the transformations, each decile's mean for each transformation was converted to a Cohen's *d* by dividing it by the transformation's *SD* for the full sample. The figure plots the mean of each transformation for each of 10 latency deciles (overall N  =  2,023, Ns per decile range from 202 to 203).

The desired form of the latency operating characteristic is flat, which would indicate that the plotted measure's sensitivity to its intended construct is unaffected by variations in speed of responding. But this expectation of a flat shape depends also on freedom from influence by some third variable that might be correlated strongly enough with *both* latency and the plotted measure. The only third variable known confidently to correlate with individual differences in latency is age (*r*  = .25 in the present sample). If, for example the plotted political attitude measure is correlated at r  = .40 with age, a true correlation of 0 between latency and the measure would be altered to + .10 ( = .25 ×.40). In the present sample, the BIAT D measure was correlated with latency at *r*  = .06, which is much too small to be responsible for any noticeable deviation from a flat LOC. Correlations of average latency with explicit political attitude measures were likewise small to be of concern (*r*  =  −.03 for liberal–conservative ideology, and *r*s =  −.02 and −.11 for two measures of preference for Republican over Democrat).

Fig. 2 shows that all four transformations were sensitive to the politically liberal character of the sample. Nevertheless, they varied considerably both in sensitivity and in stability across the 10 deciles. The most obvious deviation from stability in Fig. 2 is for the untransformed latency difference measure, which was clearly larger in value for slow than fast deciles. This was true to a lesser extent for the log transformation. The *D* transformation showed the opposite trend, being smaller for the slowest subjects. To assess stability statistically, the four transformations were entered as criteria in separate multiple regression analyses that used linear, quadratic, cubic, and quartic trends of the average latency measure for each subject as predictors. Stability is revealed by a *small* size of the Multiple *R* in this analysis. Ordered from greatest to least stability, the four transformations were *Reciprocal* (*R*  = .034, *p*  = .69), *Log* (*R*  = .052, *p*  = .24), *D* (*R*  = .076, *p*  = .02), and *Latency* (*R*  = .154, *p*  = 10^−9^).

**Figure 3:**
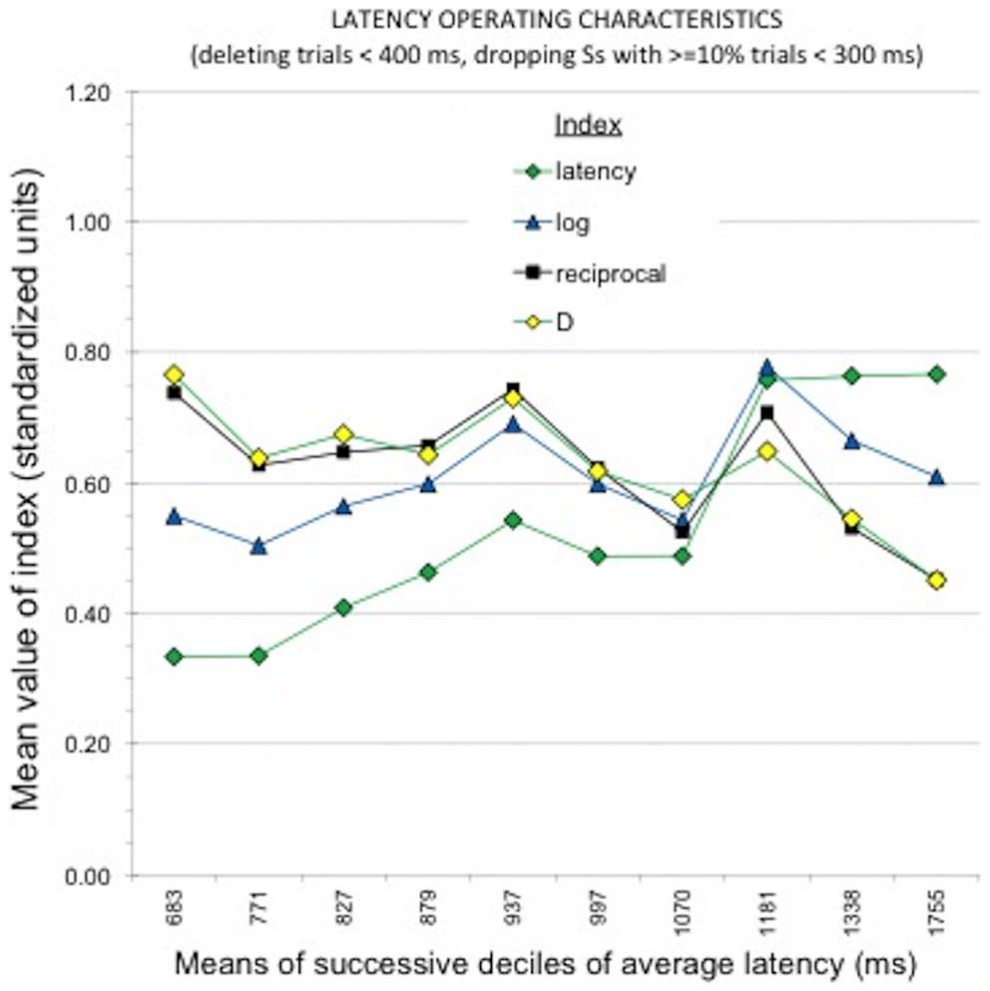
Latency operating characteristics, showing variation in mean standardized values of the five candidate BIAT scoring algorithms across deciles of the sample's distribution of average speed of responding for the political BIAT. Pretreatment of the data involved removing 4 warm-up trials per block, latencies slower than 10s, and latencies faster than 400 ms. There were 202 or 203 respondents in each decile. The most noticeable effects visible in the graph are improvement in performance of the reciprocal measure relative to its poor showing in Fig. 2, and the contrast between the relative stability across speed variations for four of the measures and the increasing magnitude of the (untransformed) latency-difference measure as responding went from fast (left of graph) to slow.

To show the influence of fast responding on the four transformations, Fig. 3 shows the same latency operating characteristics as Fig. 2, but for measures in which, additionally, latencies faster than 400 ms were deleted before computing the measure. The patterns are partly the same. The latency and logarithm transformations still show greater values for slower subjects, and the *D* measure still shows smaller values for slower subjects. The most dramatic difference is for the reciprocal transformation, which has values nearly double those in Fig. 2. Results for the polynomial regressions were very similar to those for the data in Fig. 2.

#### Sensitivity to correlation with political orientation

Latency operating characteristics using the same data selection criteria as those in Figs. 2 and 3 were also computed using as criterion measure the correlation between each BIAT transformation and the 1-item measure of conservative–liberal political orientation. Conclusions were similar to those from Figs. 2 and 3, showing that the reciprocal measure was much more sensitive to including versus dropping latencies faster than 400 ms, and the other measures performed similarly to one another, with the *D* measure outperforming the others in stability across latency variations and in larger magnitude of effect (with correlations averaging *r*  = .57).

#### Other criteria

Summary results across the other evaluation criteria with latencies faster than 400 ms deleted are summarized in the right-side panel of Table 3 for both good-focal and bad-focal response blocks (see S1 Table for results with race and S2 Table for results with self-esteem). Across criteria, *D* performed consistently strongly. *Reciprocal* also performed well in many cases, and *Log* and particularly *Latency* performing less well, albeit only slightly in some cases for *Log*.

As a summary of Study 2, *D* performed best among the four transformations. Without truncation of fast responses *Reciprocal* showed the least sensitivity to expected effects, and *Latency* showed greatest susceptibility to artifact associated with speed of responding and did not perform as well on the other criteria. *Log* was satisfactory in many respects, but was nevertheless consistently outperformed by *D*.

## Study 3: Treatment of Extreme Latencies

Because the preceding studies had made clear that *D* was the most effective measure, starting with Study 3 we changed focus to finding D′s best form. Although the reciprocal transformation was consistently third best in analyses when responses faster than 400 ms were removed, it was not considered further because its properties were quite poor without those removals (see, e.g., Fig. 2).

In speeded response tasks, very rapid and very slow responses are often treated as due to subjects deviating from instructed behavior. Study 3 examined alternative methods for reducing the influence of these outlying observations on psychometric properties of the *D* measure. Each latency-tail treatment was identified by boundary values for fast and slow responding. For each candidate boundary value, we examined effects either of removing trials outside that boundary or of recoding outlying trials to the boundary values, or both. The boundaries and strategies that were examined were: no removal, deleting below 200 or 400 ms boundaries, recoding below 400 ms to that boundary value, deleting above 2000, 3000 or 4000 ms, and recoding above 2000, 3000, or 4000 ms to those boundary values. Although the *D* measure preserves distribution information, it also reduces the impact of outlying observations by using the subject's variability as a denominator for latency differences between treatments. Outlying observations, in effect, reduce their own impact by contributing to the magnitude of the denominator.

### Results and Discussion

Analyses were conducted after deleting the first four trials of each response block and excluding participants who had more than 10% of latencies faster than 300ms. Findings are reported for good-focal BIATs (see Table 4). We also report results for replications with race (S3 Table) and self-esteem (S4 Table). For *D*, recoding with 400 and 2000 boundaries produced the best performance. Overall, the results suggest that *D* is relatively insensitive to treatments of extreme latencies.

**Table 4:** Comparison of fast and slow latency treatments across evaluation criteria for politics good focal response blocks (Study 3).

	Fast Latency Treatment								Slow Latency Treatment		
	Deleting			Recoding		Deleting			Recoding		
	D400	D200	D none	G400	D400	D400 + D2000	D400 + D3000	D400 + D4000	D400 + D2000	D400 + D3000	D400 + D4000
**KNOWN GROUP DIFFERENCE** (Political ID)	0.557	0.556	0.556	0.563	0.557	0.569	0.567	0.560	0.567	0.560	0.559
**INTERNAL CONSISTENCY** (alpha)	0.772	0.770	0.768	0.780	0.770	0.770	0.765	0.769	0.779	0.772	0.771
											
**RELATIONS WITH OTHER IMPLICIT MEASURES**											
IAT	0.645	0.643	0.641	0.640	0.643	0.654	0.653	0.657	0.648	0.645	0.643
GNAT	0.676	0.676	0.675	0.674	0.677	0.667	0.667	0.667	0.677	0.674	0.675
ST-IAT	0.607	0.607	0.606	0.603	0.607	0.622	0.612	0.604	0.612	0.605	0.605
SPF	0.619	0.611	0.610	0.593	0.611	0.622	0.616	0.620	0.611	0.610	0.609
EPT	0.561	0.559	0.559	0.558	0.560	0.561	0.554	0.553	0.556	0.558	0.559
AMP	0.484	0.482	0.482	0.495	0.482	0.515	0.494	0.496	0.491	0.483	0.482
SPD	0.617	0.621	0.620	0.626	0.621	0.610	0.614	0.613	0.624	0.621	0.621
											
Average	0.604	0.603	0.602	0.601	0.603	0.609	0.604	0.604	0.606	0.602	0.602
											
**RELATIONS WITH SELF-REPORT MEASURES AND CRITERION VARIABLES**											
Dem-Rep Preference	0.672	0.668	0.664	0.671	0.666	0.694	0.689	0.680	0.679	0.673	0.670
Warmth for Democrats	0.452	0.451	0.446	0.461	0.448	0.475	0.460	0.457	0.459	0.452	0.450
Warmth for Republicans	0.572	0.571	0.567	0.579	0.570	0.590	0.588	0.579	0.580	0.573	0.572
Right-Wing Authoritarianism	0.538	0.537	0.537	0.545	0.537	0.531	0.533	0.537	0.535	0.537	0.537
Avg liking of 5 Democrats	0.567	0.570	0.568	0.581	0.568	0.566	0.568	0.570	0.577	0.571	0.569
Avg liking of 5 Republicans	0.617	0.617	0.615	0.641	0.615	0.644	0.646	0.628	0.639	0.623	0.618
Intended Vote in 2008 (D or R cand.)	0.562	0.558	0.558	0.561	0.559	0.582	0.568	0.566	0.570	0.563	0.561
Vote in 2004 (Bush or Kerry)	0.692	0.690	0.685	0.677	0.687	0.690	0.698	0.698	0.692	0.691	0.690
											
Average	0.589	0.587	0.584	0.594	0.586	0.601	0.599	0.594	0.596	0.590	0.588
											
**RELATIONS WITH EXTRANEOUS INFLUENCE**											
Relation with average reciprocal	0.067	0.065	0.070	0.017	0.069	0.026	0.040	0.056	0.015	0.044	0.056
Relation with average log	−0.111	−0.111	−0.111	−0.052	−0.111	−0.055	−0.080	−0.094	−0.047	−0.079	−0.094
Relation with average latency	−0.111	−0.111	−0.110	−0.054	−0.111	−0.051	−0.078	−0.092	−0.046	−0.077	−0.092

## Study 4: Error Trial Treatment

When participants make a categorization error in the BIAT they must correct it before moving on to the next trial. The trial latency is the time from stimulus onset until the correct response is made. Studies 1–3 retained all trials whether or not an error occurred. Alternative analytic strategies are to remove or recode error trials before calculating BIAT scores. On the basis of evidence obtained with the IAT [7], we expected that error trials would provide useful data and that it would likely therefore be best to retain them in computing the measure.

As an aside, there are research applications using the IAT in which respondents are not required to correct errors, though that is not recommended practice [9]. We do not consider that procedural format in this manuscript. If such procedures are used, Greenwald et al. [7] should be consulted for appropriate scoring practices.

### Results and Discussion

We removed the first four trials of each response block and trials faster than 400 ms, excluded participants having more than 10% of trials with response latencies faster than 300 ms, and we summarize results for the good-focal blocks (Table 5).

**Table 5:** Comparing effects of removing versus retaining error trials for good focal blocks on evaluation criteria (Study 4).

	Politics		Race		Self-Esteem	
	Remove	Retain	Remove	Retain	Remove	Retain
MAGNITUDE OF MAIN EFFECT	.	.	0.357	0.450	1.026	1.097
KNOWN GROUP DIFFERENCES	0.528	0.557	0.178	0.192	.	.
INTERNAL CONSISTENCY	0.721	0.773	0.545	0.569	0.402	0.432
RELATIONS WITH OTHER IMPLICIT MEASURES	0.577	0.604	0.315	0.327	0.055	0.050
RELATIONS WITH CRITERION VARIABLES	0.569	0.589	0.189	0.198	0.051	0.059
RELATIONS WITH EXTRANEOUS INFLUENCE	−0.091	−0.095	0.002	−0.025	−0.044	−0.070

Notes: Magnitude of main effect is Cohen's d of average BIAT score, others are correlation coefficients. Correlation coeffecients underwent Fisher's z transformation before averaging.

We compared BIAT scores with and without error trials removed for five evaluation criteria for *D*. The political attitude BIAT was more sensitive to differences between liberals and conservatives when error trials were retained (*r*  = .557) than when they were removed (*r*  = .528). Also, internal consistency was higher with error trials retained (*α*  = .773) than when removed (*α*  = .721). The BIAT correlated more strongly with each implicit measure with error trials retained (average *r*  = .604) than with error trials removed (average *r*  = .577). Further, the BIAT correlated more strongly with 7 of 8 self-reported attitudes and other criterion variables with error trials retained (average *r*  = .589) than with error trials removed (average *r*  = .569). Removing the error trials resulted in a roughly equal strength of relationship with the average response latency extraneous influence (*r*  =  −.091) than retaining the error trials (*r*  = .095), but the direction of the relationship differed between the two treatments. These findings support retaining error trials as useful contributors to the BIAT measure.

## Study 5: Analytic Strategy–Respondent Exclusion Criteria

When participants neglect task instructions they may produce data that is relatively useless for measurement. Two available indicators of failure to perform the BIAT as instructed were responding more rapidly than is plausible for intentional, accurate responding and making frequent errors. These are correlated indicators, because subjects who respond too rapidly will also have increased error rates. Study 4 showed that error trials can provide useful data. The study that produced the currently preferred IAT scoring algorithm [7] found that subjects who had more than 10% responses faster than 300 ms (“fast” responses) provided generally useless data and were best dropped from analyses. We compared three exclusion criteria based on response speed: no exclusions and exclusions based on exceeding either 10% or 20% of responses faster than 300 ms. We also examined three exclusion criteria based on error rates: no exclusion and exclusions based on exceeding either 30% or 40% error rates.

### Results and Discussion

Results were computed using data sets from which the four warm-up responses and latencies slower than 10 s were initially removed. Results are reported for BIAT measures based on good-focal blocks. The results were similar for measures computed from bad-focal blocks (Table 6).

**Table 6:** Effects of applying task exclusion criteria on evaluation criteria for politics (Study 5).

	Exclusion for % fast trials (no error exclusion)			Exclusion for % errors (in addition to >10% fast exclusion)	
	none	20%	10%	40%	30%
N	2145	2080	2063	2037	1966
% of tasks excluded		3.0%	3.8%	1.3%	4.7%
cumulative % of tasks excluded				5.0%	8.3%
**KNOWN GROUP DIFFERENCES**					
Political Ideology (Bad Focal)	0.372	0.384	0.388	0.389	0.400
Political Ideology (Good Focal)	0.548	0.558	0.562	0.567	0.569
**INTERNAL CONSISTENCY**					
Alpha (Bad Focal)	0.585	0.602	0.604	0.604	0.612
Alpha (Good Focal)	0.763	0.775	0.780	0.782	0.783
**RELATIONS WITH OTHER IMPLICIT MEASURES**					
*BAD FOCAL*					
IAT	0.471	0.485	0.485	0.490	0.486
GNAT	0.569	0.570	0.573	0.572	0.572
ST-IAT	0.412	0.406	0.405	0.400	0.390
SPF	0.460	0.466	0.472	0.472	0.467
EPT	0.366	0.362	0.358	0.357	0.360
AMP	0.272	0.271	0.274	0.287	0.290
SPD	0.388	0.385	0.383	0.381	0.381
*GOOD FOCAL*					
IAT	0.638	0.640	0.640	0.649	0.653
GNAT	0.665	0.673	0.674	0.674	0.676
ST-IAT	0.609	0.603	0.602	0.604	0.595
SPF	0.592	0.592	0.592	0.592	0.592
EPT	0.559	0.560	0.558	0.558	0.557
AMP	0.497	0.495	0.495	0.504	0.504
SPD	0.626	0.626	0.625	0.627	0.625
Bad focal average	0.424	0.425	0.426	0.427	0.425
Good focal average	0.600	0.601	0.601	0.604	0.603
**RELATIONS WITH SELF-REPORT MEASURES AND CRITERION VARIABLES**					
*BAD FOCAL*					
Dem-Rep Preference	0.431	0.437	0.437	0.434	0.428
Warmth for Democrats	0.335	0.348	0.350	0.348	0.356
Warmth for Republicans	0.394	0.401	0.401	0.399	0.389
Right-Wing Authoritarianism	0.468	0.466	0.467	0.464	0.464
Avg liking of 5 Democrats	0.332	0.344	0.344	0.351	0.352
Avg liking of 5 Republicans	0.368	0.382	0.382	0.377	0.372
Intended Vote in 2008 (D or R cand.)	0.323	0.334	0.334	0.339	0.333
Vote in 2004 (Bush or Kerry)	0.456	0.458	0.452	0.452	0.452
*GOOD FOCAL*					
Dem-Rep Preference	0.655	0.671	0.671	0.670	0.677
Warmth for Democrats	0.445	0.465	0.461	0.458	0.473
Warmth for Republicans	0.560	0.570	0.579	0.576	0.572
Right-Wing Authoritarianism	0.541	0.545	0.544	0.548	0.548
Avg liking of 5 Democrats	0.571	0.581	0.581	0.579	0.581
Avg liking of 5 Republicans	0.634	0.641	0.641	0.637	0.639
Intended Vote in 2008 (D or R cand.)	0.538	0.556	0.561	0.557	0.560
Vote in 2004 (Bush or Kerry)	0.618	0.660	0.676	0.673	0.669
Bad focal average	0.390	0.397	0.397	0.397	0.394
Good focal average	0.573	0.590	0.593	0.591	0.594
**RELATIONS WITH EXTRANEOUS INFLUENCE**					
*BAD FOCAL*					
Relation with average reciprocal	−0.072	0.047	0.073	0.070	0.075
Relation with average log	−0.010	−0.106	−0.107	−0.100	−0.102
Relation with average latency	−0.069	−0.103	−0.104	−0.096	−0.100
*GOOD FOCAL*					
Relation with average reciprocal	−0.092	0.001	0.019	0.018	0.022
Relation with average log	0.047	−0.049	−0.053	−0.048	−0.050
Relation with average latency	−0.015	−0.054	−0.054	−0.047	−0.049

**Figure 4:**
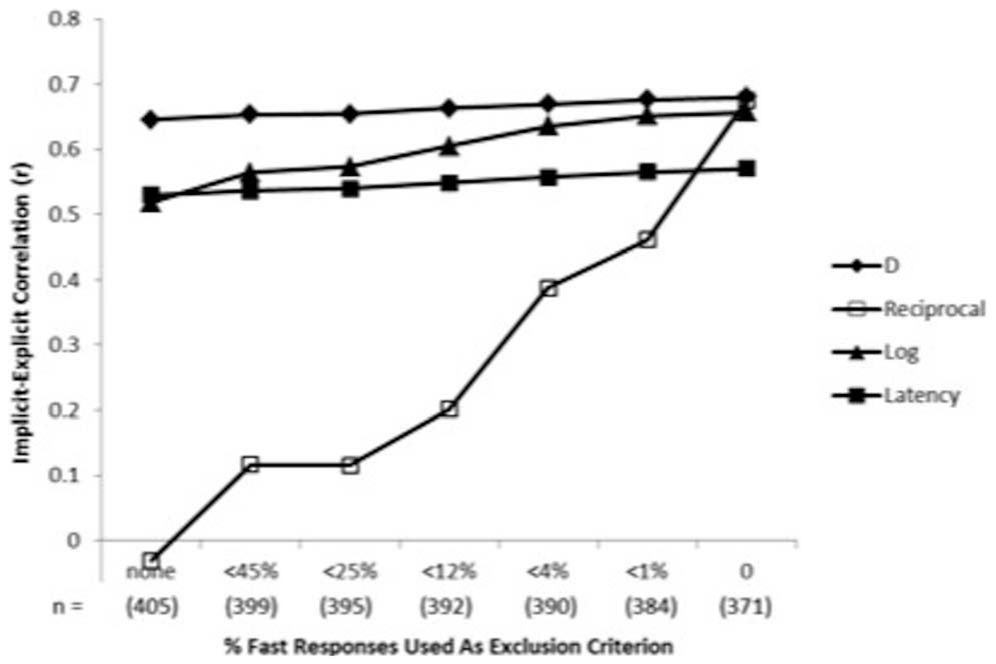
Effects of seven criteria for excluding respondents as a function of their proportion of fast responses (latency <300 ms) on correlations with self-reported preference between Democrats and Republicans for five BIAT data transformations (Study 5). Higher correlations indicate better performance. The furthest left datapoint indicates no exclusion of participants; the furthest right datapoint indicates exclusion of all participants that had even a single fast response. Sample size (n) on the x-axis indicates the number of participants retained with that exclusion criterion.

We examined the exclusion criteria sequentially – first comparing the fast trial exclusion rules, and then comparing the error rate exclusion rules. As was previously found for the IAT, excluding subjects with more than 10% fast responses (3.8% of subjects) produced psychometric benefits superior to either no exclusion or to the 20% criterion which excluded fewer (3.0% of subjects). The 10%-fast-response exclusion criterion produced best psychometric properties for detecting known group differences, for internal consistency, for correlations with parallel self-report attitude (see Fig. 4) and other criterion variables, for correlations with parallel implicit measures, and for freedom from contamination by variations in average latency of responding.

Starting from the base of excluding subjects with more than 10% of “fast” responses, the 40% error criterion eliminated another 1.3% of the sample (5.0% excluded in total), while the 30% error criterion eliminated 4.7% of the sample (8.3% excluded in total). The 30% exclusion criterion afforded greater sensitivity to known group differences and slightly stronger internal consistency compared to no error-based exclusion, but did not improve over no extra error-based exclusion for relations with implicit measures, relations with self-reported attitudes and criterion variables, and freedom from contamination by variations in average latency of responding.

We replicated these results with racial attitudes and self-esteem (see S1 File). The results suggest that excluding subjects with more than 10% fast responses has benefits for overall psychometric performance. Because the 10%-fast-responding criterion effectively excludes most subjects who have higher error rates, additional exclusion of remaining subjects with 30% or more errors has only a small additional beneficial effect. Nevertheless, it is possible that the 30%-error-rate exclusion criterion will prove useful in small samples because of the stronger impact of single, outlier scores on measurement performance. Further, note that these exclusion criteria are for the psychometric evaluation of the sample as a whole. Interpretation of single scores may require distinct criteria.

## Study 6: Comparing Good-Focal and Bad-Focal Blocks

Sriram and Greenwald [8] observed that response blocks in which *Good words* was a focal category and *Bad words* was nonfocal showed more reliable effects and stronger correlations with criterion variables than did those in which *Bad words* was focal and *Good words* was nonfocal. This is a curious phenomenon because the two variations are structurally identical. In both cases, there are two response blocks: In the political BIAT (a) in one block *Democrats* and *Good words* are categorized with one key and *Republican* and *Bad words* are categorized with the other key, and (b) in the other block *Republicans* and *Good words* are categorized with one key and *Democrats* and *Bad words* with the other key. The only difference between the good-focal and bad-focal conditions is in the category labels that appear on screen and to which participants are instructed to attend. In the “good-focal” condition, the category labels appear as “Democrats and Good” and “Republicans and Good” naming the two categories required for one of the responses keys in the respective response blocks described above. Respondents are instructed to categorize “anything else” with the second key. In the “bad-focal” condition, the category labels appear as “Republicans and Bad” and “Democrats and Bad” for the same response blocks. Sriram and Greenwald's finding that good-focal and bad-focal conditions elicit different degrees of validity was intriguing and important to clarify. Study 6 sought to examine this phenomenon with a variety of evaluation criteria. The results demonstrate the procedural advantage of using “good” as the focal attribute for attitude BIATs.

### Results and Discussion

We applied the same data preparation practices as in Study 2 adding the exclusion of latencies faster than 400 ms, and – because the results were already available - we compared focal conditions on all four candidate data transformation approaches from that study. We compared good-focal and bad-focal conditions on (a) sensitivity to known group differences, (b) internal consistency, (c) relations with other implicit measures, and (d) relations with parallel self-report measures and criterion variables.

We compared the correlation of the political attitudes BIAT with self-reported political orientation between the two focal conditions. Across the four data transformation procedures, political orientation was more strongly correlated with the good-focal BIAT (average *r*  = .527; range among scoring transformations .457 to .557) than with the bad-focal BIAT (average *r*  = .355; range .282 to .393; see Table 3). In other words, political ideology accounted for almost 200% more shared variance in the good-focal BIAT (27.8%) than in the bad-focal BIAT (12.6%) despite them being structurally identical. Likewise, the good-focal BIAT showed much stronger internal consistency (average α  = .753; range .690 to .778) than did the bad-focal BIAT (average α  = .538; range .389 to .623). Further, the good-focal BIAT correlated more strongly (average *r*  = .563; range .477 to .604) with seven other implicit measures of political attitudes than did the bad-focal BIAT (average *r*  = .383; range .293 to .428). Finally, the good-focal BIAT correlated more strongly (average *r*  = .558; range .491 to .589) with eight self-reported criterion variables such as past voting and voting intention than did the bad-focal BIAT (average *r*  = .364; range .292 to .398). These differences indicate sizable internal consistency and validity advantages for the good-focal over the bad-focal conditions.

We replicated the comparison of good and bad-focal blocks with racial attitude measures, and with self-esteem measures (see S1 and S2 Tables). The results consistently replicated for racial attitudes, and offer the same conclusion but somewhat less definitively for self-esteem. In particular, the self-esteem BIAT showed weak relations with other implicit measures and with the criterion variables for both good and bad-focal blocks [14]; [37]. On the other criteria, good-focal retained a clear advantage. These results suggest that attitude BIATs may be much more effective by using *good* as the constant focal category and *bad* as the constant non-focal category. In additional laboratory data, there is a similar advantage for using *self* as the focal category instead of *other* for identity-related IATs [8]. Identification of the mechanism underlying these differences may assist in selecting focal and background categories for other applications. A qualification of this conclusion is the possibility that *bad* and *other* focal blocks reveal distinct validity, even though their psychometric performance is weaker overall. As such, there may be many research applications in which collecting data for both focal conditions is theoretically relevant and advisable.

## Study 7: First 40 vs. Second 40 Trials

In the development of the IAT scoring algorithm, data from the first blocks of each combined task produced a measure slightly superior to that from the second blocks of each task [7]. In the present research, each of the good-focal and bad-focal BIATs was conducted in four blocks, producing one measure for the first two blocks and another for the second two. These two sub-measures provided the basis for previously described internal consistency tests.

For Study 7′s comparisons of the two sub-measures we used the best performing versions of the *D* measure — ones computed from data sets for which 4 warm-up trials of each block and latencies greater than 10 s had been removed, and for which latencies faster than 400 ms and slower than 2000 ms had been recoded to those boundary values. Also, data for subjects having more than 10% of responses faster than 300 ms were excluded. These *D* measures were compared in their sensitivity to the liberal character of the subject population, and their average correlations with the self-reported and implicit political attitude measures, and also their (weaker) average correlations with three self-report race attitude measures and with seven available implicit race attitude measures.

### Results and Discussion

Table 7 compares properties of *D* measures based on first 40 trials versus second 40 trials of the political BIAT measure. Each set of 40 trials consisted of two 20-trial blocks, one with Democrat and good focal, the other with Republican and good focal. Results for the *D* measure computed without any latency tail treatments are included in Table 7 for comparison.

**Table 7:** Analyses of *D* measure based on First 40 Trials vs. Second 40 Trials.

	Recoding	Trial subsets	BIAT	*r*_explicit political_	*r*_explicit race_	*r*_implicit political_	*r*_implicit race_	Cron-bach α
	none	1st 40	0.583	.543	.248	.543	.152	.768
D		2nd 40	0.558	.581	.241	.562	.170	
	<400 = 400;	1st 40	0.591	.559	.249	.549	.154	.779
	>2000 = 2000	2nd 40	0.567	.590	.245	**.**563	.173	

Note. Recoding treatments are described in text. *D* is the best performing BIAT scoring algorithm as described in the text. Underlines indicate the trial subset (1st or 2nd) with larger value for each combination of measure type and recoding treatment. The “BIAT” column gives Cohen's d effect size measures for difference of mean BIAT scores from zero. *r*_explicit.political_ is the averaged correlation of the political BIAT with seven self-report measures of political beliefs (range of Ns: 229–2,057); *r*_explicit.race_ is the averaged correlation of the political BIAT with three self-report measures of racial attitudes (range of Ns: 446–463); *r*_implicit.political_ is the BIAT's average correlation with 7 other implicit political measures (range of Ns: 255–435); *r*_implicit.race_ is the BIAT's average correlation with 7 implicit measures of race attitudes (range of Ns: 256–425); Cronbach's α is a measure of internal consistency based on each pair of 40-trial measures (N = 2,136).

The most striking feature of the results in Table 7 were that average correlations with political measures were quite substantial for *D*, regardless of whether the measures used tail treatments or not and whether they were based on the first 40 or last 40 trials. Although the averaged correlations with self-report and implicit race attitude measures were lower, all of the individual correlations with explicit race attitude measures were statistically significant, and most of those with implicit measures were likewise statistically significant. Additionally, the internal consistency data showed Cronbach's alphas close to .80.

Those observations, however, do not bear on the main reason for interest in these data, which were to determine whether there was a difference between the two sets of 40 trials in their sensitivity to expected effects.

In fact, the data provide no clear basis for preferring measures based on the first 40 or the second 40 trials of each BIAT. And, both sets of trials contribute to measurement validity with the first 40 performing best on a few criteria and the second 40 on other criteria. In examining these data in conjunction with replications using the race and self-esteem BIATs, there are indications that *D* measures consistently showed small benefits of tail treatments. It may require data sets with considerably more observations than even the large data set of the present research to establish the generalizability of these observations.

## General Discussion

The present studies identified analytic practices that maximized (a) sensitivity to known effects and group differences, (b) internal consistency, (c) relations with other implicit measures of the same topic, (d) relations with self-report measures of the same topic and other criterion variables, and (e) resistance to the extraneous influence of average response time for the Brief Implicit Association Test. The studies and replications showed that (a) the four warm-up trials at the beginning of each response block do not contribute to the measures' validity (Study 1), (b) the *D* data transformation performs better than variations that use differences between average response times (Study 2), (c) trials in which an error is made provide useful information and should be retained in analysis (Study 3), (d) task performances with a high frequency of unreasonably fast responses and high error rates (to a lesser degree) may be removed to improve overall sensitivity and measure performance (Study 4), (e) treatment of extreme latencies has relatively small effects, but can improve *D* slightly by either recoding or removing very fast and very slow trials (Study 5), (f) “good-focal” response blocks possess much stronger psychometric properties than “bad-focal” response blocks (Study 6), and (g) the first and second halves of the BIAT contributed approximately equally to the measures' validity (Study 7). These findings converge to recommended BIAT analytic practices that are presented in Table 8. Future research may identify additional improvements among the variations.

**Table 8:** Recommended scoring practice for BIAT using procedure described in Table 1.

	Steps for scoring with D
1	Remove trials >10000 milliseconds
2	Remove 1st four trials of each response block
3	Retain error trials
4	Recode <400 ms to 400 ms and >2000 ms to 2000 ms
5	Compute D separately for each pair of two consecutive blocks separately, and then average
6	Remove tasks with >10% fast responses

The results also replicated and extend Greenwald and colleagues' [7] observation that *D* strongly outperformed conventional data transformation techniques for analysis of the IAT. The present results showed that *D*′s value extended to the BIAT and also demonstrated notable improvements in a variety of additional criteria including the strength of correlations with other implicit measures. The present studies had the advantage of evaluating the algorithms with known findings, large samples, and multiple replications allowing for inference based on algorithm performance. In typical research applications using the BIAT, especially when the hypothesized outcome is not already known to exist, selection of scoring algorithm should occur prior to data analysis, not following analysis based on which one elicited the best performance in that particular dataset. Applying standard analytic practices will facilitate comparison of effects across research applications. Instructions for calculating the recommended D scoring procedure appears in Table 2.

The recommended scoring practices were the best performing in these studies, but that does not mean that these are the best possible scoring practices in general. Future investigation of alternative scoring algorithms may reveal improvements, both for general application as well as for heightened sensitivity to specific populations or manipulations. However, in those cases where the scoring algorithm itself is not the subject of investigation, maximum credibility and comparability is achieved by following default scoring practices rather than making idiosyncratic scoring selections based on which “look best” for a particular research application.

### Implications and future directions

#### Good primacy

“Good-focal” blocks displayed a performance advantage over “bad-focal” blocks despite being behaviorally identical. Participants are using the same keys, with the same response assignments, and categorizing the same stimuli. The only difference are the instructions for what information to attend to, and the labels that appear on screen. This illustrates the significant impact instructions and the mental context can have on implicit measurement, and it offers an intriguing puzzle that neither Sriram and Greenwald [8] nor we have the evidence to solve. Unkelbach and colleagues [38] proposed that positive information is more similar to other positive information and is more strongly associated with other positive information, in comparison to the similarity and association that negative information share with other negative information [38]. It is conceivable that this could contribute to good primacy in the BIAT.

Previous research may also be able to shed light on this asymmetry for good-focal and bad-focal performance. Salience asymmetries are a potential influence on IAT effects [39]–[41], and could likewise influence BIAT effects. Similarly, good and bad-focal BIATs may have differential recoding costs, leading to diverging results [42]. The differences between good and bad-focal BIATs merit more investigation.

#### Potential applicability of D to other procedures

*D* was developed for analysis of response latency data in the contrasting conditions of the IAT and BIAT. However, it has the potential for much broader application. Sriram, Nosek, and Greenwald [43] propose scale invariance and validity maximization as defining properties of admissible latency contrasts, and perhaps for other measures that have mean-variance correlations (i.e., differences in means between conditions are associated with differences in variances between conditions). In addition, there is potential for further developments in improving research efficiency with optimal algorithms through the creation and refinement of new scoring methods and simulations [23]; [43].

## Conclusion

Research efficiency – the amount of knowledge gained compared to the resources expended – is improved by maximizing the validity of measurement methods. In the present article, we identified analytic practices that improve the validity of the BIAT. Applying these practices, and adapting further improvements when they are identified, will accelerate the discovery of the relevance of implicit cognition for human behavior.

## Supporting Information

S1 File **Across three topics, there is no block order effect in the Brief Implicit Association Test (BIAT) with an ABAB block design.**
(PDF)

S1 Table **Comparison of bad and good focal blocks, retaining or removing 1st four trials of each block, and candidate data transformations on evaluation criteria for racial attitudes.** Magnitude of main effect is Cohen's d of average BIAT score, others are correlation coefficients. Correlations averaged after Fisher's z-transformation and then converted back to a correlation.
(DOCX)

S2 Table **Comparison of bad and good focal blocks, retaining or removing 1st four trials of each block, and candidate data transformations on evaluation criteria for self-esteem.** Magnitude of main effect is Cohen's d of average BIAT score, others are correlation coefficients. Correlations averaged after Fisher's z-transformation and then converted back to a correlation.
(DOCX)

S3 Table **Comparison of fast and slow latency treatments across evaluation criteria for race.** Magnitude of main effect is Cohen's d of average BIAT score, others are correlation coefficients. Correlations averaged after Fisher's z-transformation and then converted back to a correlation.
(DOCX)

S4 Table **Comparison of fast and slow latency treatments across evaluation criteria for self-esteem.** Magnitude of main effect is Cohen's d of average BIAT score, others are correlation coefficients. Correlations averaged after Fisher's z-transformation and then converted back to a correlation.
(DOCX)
