# Supplementary material for: Understanding and Using the Brief Implicit Association Test: Recommended Scoring Procedures
Source: PLoS One. 2014 Dec 8;9(12):e110938. doi: 10.1371/journal.pone.0110938 (PMC4259300; doi:10.1371/journal.pone.0110938)
Supplement: S1 File — Across three topics, there is no block order effect in the Brief Implicit Association Test (BIAT) with an ABAB block design. (PDF) [file pone.0110938.s001.pdf]

Supplement to Nosek, Bar-Anan, Sriram, Axt and Greenwald (2014) Procedural Test

Summary: Across three topics, there is no block order effect in the Brief Implicit Association Test (BIAT) with an ABAB block design

One of the most persistent extraneous influences with comparative response latency tasks is the order of presentation. Response blocks performed first tend to interfere with response blocks performed next. This order effect is well known with the IAT (Greenwald et al., 1998), and a procedural innovation was introduced to reduce its influence (Nosek et al., 2005). In this large-scale data collection, we did not experimentally manipulate factors to address the order effect, but we did introduce a study design that we hypothesized might eliminate it. Participants completed a brief warm-up block for 16 trials with irrelevant stimuli (Mammals and Good words as focal, Birds and Bad words were non focal), and then the two critical response blocks in an ABAB design. For example, if the participant received "Democrats and Good" first, then "Republicans and Good" would occur second, followed by a repetition of this sequence in blocks 3 and 4. In this case, "bad-focal" blocks would then occur as blocks 5-8.

Additionally, the procedural design randomized whether "good-focal" or "bad-focal" blocks occurred first. It is possible that this procedural factor likewise had an impact on BIAT scores with the later block producing overall weaker scores than the earlier block due to familiarization with the procedure and practice (Greenwald et al., 2003). In this analysis, we evaluated whether either of these procedural factors influenced BIAT scores across the five candidate data transformations from the primary article. If they did not, then the ABAB procedural design would be preferred to alternatives that might produce the extraneous influence of order.

Results and Discussion

Based on prior research, we excluded participants that had >10% of trials with response latencies <300ms (Greenwald et al., 2003), and we dropped individual trials <400ms and the first four response trials of each response block (Sriram & Greenwald, 2009). These session and trial exclusion rules are evaluated formally in later studies. Also, because of the large sample sizes, multiple comparisons, and approach of different analysis strategies on the same data, the emphasis is on effect size rather than significance testing.

The block order was randomized with a 2 (good-focal first, bad-focal first) X 2 (democrats with good first, republicans with good first) design. We evaluated the effect of these two factors on BIAT measures derived from the five candidate scoring transformations (G, D, difference of mean reciprocals, difference of mean log latencies, and difference of mean latencies) separately for good and bad-focal conditions. This produced ten 2 (focal attribute in blocks 1-4: good, bad) X 2 (focal category in blocks 1-2 and 5-6: Democrats, Republicans) ANOVAs. Despite extremely high power, in just one of the 10 ANOVAs was the overall model significant, using the reciprocal score on bad-focal blocks ($F[3,2024] = 2.62$, $p =$

$.049$, $R^2 = .004$). Its tiny size and the lack of effect across the other four scoring

transformation suggests that it is a false positive. In addition, we repeated these ANOVAs and found similar results after having calculated the absolute value of the BIAT effects to test whether the order effects influenced the size of the BIAT effect (distance from zero) regardless of which response block was faster. Overall, the results indicate that the procedural order manipulations had no impact on the resulting BIAT effects (all other $R^2 < .0030$).

We replicated the lack of influence of block order with racial attitude measures (all R^2s < .0025), and with self-esteem measure (all R^2s < .0020). The procedural order manipulations had no impact in either study. These results suggest that the ABAB block design may avoid the persistent effect of task order that can influence implicit measures such as the IAT. However, note that there was not an experimental test of alternative procedural designs. So, this conclusion is based on the comparison with prior evidence of the persistent response order effect (Nosek et al., 2007). It is possible that alternative procedures will show equal resistance to the order effect, and even better performance on other psychometric criteria.

References

Greenwald, A. G., McGhee, D. E., & Schwartz, J. K. L. (1998). Measuring individual differences in implicit cognition: The Implicit Association Test. *Journal of Personality and Social Psychology, 74*, 1464- 1480.

Greenwald, A. G., Nosek, B. A., & Banaji, M. R. (2003). Understanding and using the Implicit Association Test: I. An improved scoring algorithm. *Journal of Personality and Social Psychology*, *85*(2), 197- 216.

Nosek, B. A., Greenwald, A. G., & Banaji, M. R. (2005). Understanding and using the Implicit Association Test: II. Method variables and construct validity. *Personality and Social Psychology Bulletin, 31*(2), 166-180.

Nosek, B. A., Greenwald, A. G., & Banaji, M. R. (2007). The Implicit Association Test at age 7: A methodological and conceptual review. In J. A. Bargh (Ed.), *Social Psychology and the Unconscious: The Automaticity of Higher Mental Processes* (pp. 265-292). New York: Psychology Press.

Sriram, N., & Greenwald, A. G. (2009). The Brief Implicit Association Test. *Experimental Psychology*, *56*, 283–294.
