# Supplementary material for: Understanding and Using the Brief Implicit Association Test: Recommended Scoring Procedures
Source: PLoS One. 2014 Dec 8;9(12):e110938. doi: 10.1371/journal.pone.0110938 (PMC4259300; doi:10.1371/journal.pone.0110938)
Supplement: S1 Table — Comparison of bad and good focal blocks, retaining or removing 1st four trials of each block, and candidate data transformations on evaluation criteria for racial attitudes. Magnitude of main effect is Cohen's d of average BIAT score, others are correlation coefficients. Correlations averaged after Fisher's z-transformation and then converted back to a correlation. (DOCX) [file pone.0110938.s002.docx]

Table S1. Comparison of bad and good focal blocks, retaining or removing 1st four trials of each block, and candidate data transformations on evaluation criteria for racial attitudes. Magnitude of main effect is Cohen's d of average BIAT score, others are correlation coefficients. Correlations averaged after Fisher's z-transformation and then converted back to a correlation.

|  |  | Retain all trials | | | | Remove 1st four trials of each block | | | | Average across algorithms | |
| --- | --- | --- | --- | --- | --- | --- | --- | --- | --- | --- | --- |
|  | N | D | Reciprocal Diff | Log Diff | Latency Diff | D | Reciprocal Diff | Log Diff | Latency Diff | All Trials | Remove 1st four |
| **MAGNITUDE OF MAIN EFFECT** | | | | | | | | | | | |
| Preference for White (Bad Focal) | 2232 | 0.572 | 0.589 | 0.562 | 0.425 | 0.692 | 0.666 | 0.65 | 0.522 | 0.585 | 0.637 |
| Preference for White (Good Focal) | 2230 | 0.409 | 0.417 | 0.408 | 0.338 | 0.450 | 0.442 | 0.438 | 0.376 | 0.393 | 0.427 |
| **KNOWN GROUP DIFFERENCES** |  |  |  |  |  |  |  |  |  |  |  |
| Participant Race - Black or White (Bad Focal) | 1783 | 0.085 | 0.078 | 0.072 | 0.048 | 0.099 | 0.090 | 0.086 | 0.060 | 0.071 | 0.084 |
| Participant Race - Black or White (Good Focal) | 1783 | 0.173 | 0.178 | 0.179 | 0.155 | 0.184 | 0.178 | 0.187 | 0.183 | 0.171 | 0.183 |
| Political Ideology (Bad Focal) | 2141 | 0.066 | 0.066 | 0.060 | 0.045 | 0.079 | 0.079 | 0.070 | 0.047 | 0.059 | 0.069 |
| Political Ideology (Good Focal) | 2141 | 0.194 | 0.217 | 0.215 | 0.187 | 0.199 | 0.210 | 0.209 | 0.183 | 0.203 | 0.200 |
| **INTERNAL CONSISTENCY** |  |  |  |  |  |  |  |  |  |  |  |
| Alpha (Bad Focal) | 2012 | 0.365 | 0.413 | 0.406 | 0.359 | 0.373 | 0.418 | 0.401 | 0.345 | 0.386 | 0.385 |
| Alpha (Good Focal) | 2024 | 0.562 | 0.609 | 0.592 | 0.518 | 0.569 | 0.592 | 0.573 | 0.492 | 0.571 | 0.558 |
| **RELATIONS WITH OTHER IMPLICIT MEASURES** | | | | | | | | | | | |
| *BAD FOCAL* |  |  |  |  |  |  |  |  |  |  |  |
| IAT | 293 | 0.329 | 0.365 | 0.323 | 0.209 | 0.399 | 0.411 | 0.374 | 0.264 | 0.308 | 0.363 |
| GNAT | 316 | 0.228 | 0.240 | 0.210 | 0.136 | 0.249 | 0.252 | 0.220 | 0.136 | 0.204 | 0.215 |
| ST-IAT | 296 | 0.232 | 0.266 | 0.259 | 0.198 | 0.253 | 0.275 | 0.270 | 0.215 | 0.239 | 0.253 |
| SPF | 304 | 0.214 | 0.194 | 0.155 | 0.097 | 0.193 | 0.197 | 0.147 | 0.066 | 0.165 | 0.151 |
| EPT | 286 | 0.056 | 0.100 | 0.092 | 0.074 | 0.055 | 0.095 | 0.078 | 0.051 | 0.081 | 0.070 |
| AMP | 399 | 0.076 | 0.106 | 0.111 | 0.102 | 0.096 | 0.103 | 0.101 | 0.087 | 0.099 | 0.097 |
| SPD | 425 | 0.070 | 0.127 | 0.098 | 0.054 | 0.118 | 0.152 | 0.122 | 0.075 | 0.087 | 0.117 |
|  |  |  |  |  |  |  |  |  |  |  |  |
| *GOOD FOCAL* |  |  |  |  |  |  |  |  |  |  |  |
| IAT | 293 | 0.399 | 0.436 | 0.421 | 0.347 | 0.404 | 0.458 | 0.447 | 0.388 | 0.401 | 0.425 |
| GNAT | 316 | 0.386 | 0.380 | 0.378 | 0.352 | 0.358 | 0.365 | 0.365 | 0.342 | 0.374 | 0.358 |
| ST-IAT | 296 | 0.328 | 0.406 | 0.374 | 0.287 | 0.375 | 0.413 | 0.385 | 0.311 | 0.350 | 0.372 |
| SPF | 304 | 0.286 | 0.283 | 0.242 | 0.158 | 0.312 | 0.305 | 0.268 | 0.196 | 0.243 | 0.271 |
| EPT | 286 | 0.303 | 0.312 | 0.290 | 0.335 | 0.308 | 0.318 | 0.299 | 0.241 | 0.310 | 0.292 |
| AMP | 399 | 0.217 | 0.221 | 0.218 | 0.179 | 0.227 | 0.224 | 0.227 | 0.203 | 0.209 | 0.220 |
| SPD | 425 | 0.293 | 0.326 | 0.314 | 0.264 | 0.297 | 0.327 | 0.316 | 0.276 | 0.299 | 0.304 |
|  |  |  |  |  |  |  |  |  |  |  |  |
| Bad focal average |  | 0.174 | 0.202 | 0.180 | 0.125 | 0.197 | 0.215 | 0.190 | 0.129 | 0.170 | 0.183 |
| Good focal average |  | 0.317 | 0.340 | 0.321 | 0.276 | 0.327 | 0.346 | 0.331 | 0.281 | 0.314 | 0.322 |
| **RELATIONS WITH SELF-REPORT MEASURES AND CRITERION VARIABLES** | | | | | | | | | | | |
| *BAD FOCAL* |  |  |  |  |  |  |  |  |  |  |  |
| Black-White Preference | 487 | 0.149 | 0.129 | 0.135 | 0.126 | 0.140 | 0.139 | 0.152 | 0.149 | 0.135 | 0.145 |
| Warmth for Blacks | 503 | 0.052 | 0.058 | 0.054 | 0.039 | 0.192 | 0.081 | 0.087 | 0.080 | 0.051 | 0.110 |
| Warmth for Whites | 503 | 0.068 | 0.059 | 0.053 | 0.040 | 0.034 | 0.043 | 0.034 | 0.023 | 0.055 | 0.034 |
| Avg liking of 5 Black people | 343 | 0.017 | 0.071 | 0.051 | 0.011 | 0.072 | 0.107 | 0.090 | 0.043 | 0.038 | 0.078 |
| Avg liking of 5 White people | 342 | 0.085 | 0.114 | 0.130 | 0.126 | 0.110 | 0.124 | 0.143 | 0.146 | 0.114 | 0.131 |
| Modern Racism Scale | 484 | 0.135 | 0.119 | 0.102 | 0.080 | 0.134 | 0.140 | 0.114 | 0.075 | 0.109 | 0.116 |
| Contact with Black people | 499 | 0.104 | 0.076 | 0.090 | 0.103 | 0.091 | 0.092 | 0.106 | 0.113 | 0.093 | 0.101 |
| Right-Wing Authoritarianism | 453 | 0.095 | 0.141 | 0.115 | 0.067 | 0.120 | 0.154 | 0.137 | 0.098 | 0.105 | 0.127 |
|  |  |  |  |  |  |  |  |  |  |  |  |
| *GOOD FOCAL* |  |  |  |  |  |  |  |  |  |  |  |
| Black-White Preference | 487 | 0.282 | 0.278 | 0.282 | 0.243 | 0.278 | 0.274 | 0.285 | 0.272 | 0.271 | 0.277 |
| Warmth for Blacks | 503 | 0.133 | 0.129 | 0.136 | 0.138 | 0.115 | 0.114 | 0.118 | 0.115 | 0.134 | 0.116 |
| Warmth for Whites | 503 | 0.156 | 0.152 | 0.147 | 0.115 | 0.163 | 0.155 | 0.152 | 0.133 | 0.143 | 0.151 |
| Avg liking of 5 Black people | 343 | 0.131 | 0.156 | 0.161 | 0.152 | 0.133 | 0.158 | 0.162 | 0.153 | 0.150 | 0.152 |
| Avg liking of 5 White people | 342 | 0.167 | 0.200 | 0.199 | 0.184 | 0.206 | 0.212 | 0.217 | 0.216 | 0.188 | 0.213 |
| Modern Racism Scale | 484 | 0.314 | 0.363 | 0.331 | 0.257 | 0.334 | 0.357 | 0.336 | 0.281 | 0.317 | 0.327 |
| Contact with Black people | 499 | 0.094 | 0.091 | 0.087 | 0.070 | 0.111 | 0.097 | 0.099 | 0.094 | 0.086 | 0.100 |
| Right-Wing Authoritarianism | 453 | 0.253 | 0.255 | 0.282 | 0.282 | 0.236 | 0.247 | 0.271 | 0.277 | 0.268 | 0.258 |
|  |  |  |  |  |  |  |  |  |  |  |  |
| Bad focal average |  | 0.088 | 0.096 | 0.091 | 0.074 | 0.112 | 0.110 | 0.108 | 0.091 | 0.087 | 0.105 |
| Good focal average |  | 0.192 | 0.205 | 0.205 | 0.181 | 0.198 | 0.203 | 0.206 | 0.194 | 0.196 | 0.200 |
| **RELATIONS WITH EXTRANEOUS INFLUENCE** | | | | | | | | | | | |
| *BAD FOCAL* |  |  |  |  |  |  |  |  |  | average of absolute values | |
| Relation with average reciprocal | 2232 | -0.011 | 0.090 | -0.111 | -0.306 | 0.023 | 0.121 | -0.112 | -0.345 | -0.087 | -0.082 |
| Relation with average log | 2232 | 0.051 | -0.058 | 0.215 | 0.478 | 0.008 | -0.081 | 0.184 | 0.459 | 0.181 | 0.151 |
| Relation with average latency | 2232 | 0.055 | -0.028 | 0.241 | 0.519 | 0.015 | -0.051 | 0.211 | 0.506 | 0.209 | 0.182 |
|  |  |  |  |  |  |  |  |  |  |  |  |
| *GOOD FOCAL* |  |  |  |  |  |  |  |  |  | average of absolute values | |
| Relation with average reciprocal | 2230 | -0.003 | 0.025 | -0.146 | -0.290 | 0.019 | 0.055 | -0.147 | -0.342 | -0.106 | -0.107 |
| Relation with average log | 2230 | -0.001 | -0.013 | 0.210 | 0.412 | -0.018 | -0.036 | 0.185 | 0.411 | 0.158 | 0.142 |
| Relation with average latency | 2230 | -0.006 | 0.005 | 0.225 | 0.441 | -0.025 | -0.022 | 0.195 | 0.435 | 0.174 | 0.153 |
