# Supplementary material for: Understanding and Using the Brief Implicit Association Test: Recommended Scoring Procedures
Source: PLoS One. 2014 Dec 8;9(12):e110938. doi: 10.1371/journal.pone.0110938 (PMC4259300; doi:10.1371/journal.pone.0110938)
Supplement: S2 Table — Comparison of bad and good focal blocks, retaining or removing 1st four trials of each block, and candidate data transformations on evaluation criteria for self-esteem. Magnitude of main effect is Cohen's d of average BIAT score, others are correlation coefficients. Correlations averaged after Fisher's z-transformation and then converted back to a correlation. (DOCX) [file pone.0110938.s003.docx]

Table S2. Comparison of bad and good focal blocks, retaining or removing 1st four trials of each block, and candidate data transformations on evaluation criteria for self-esteem. Magnitude of main effect is Cohen's d of average BIAT score, others are correlation coefficients. Correlations averaged after Fisher's z-transformation and then converted back to a correlation.

|  |  | Retain all trials | | | | Remove 1st four trials of each block | | | | Average across algorithms | |
| --- | --- | --- | --- | --- | --- | --- | --- | --- | --- | --- | --- |
|  | N | D | Reciprocal Diff | Log Diff | Latency Diff | D | Reciprocal Diff | Log Diff | Latency Diff | All Trials | Remove 1st four |
| **MAGNITUDE OF MAIN EFFECT** | | | | | | | | | | | |
| Self-Esteem (Bad Focal) | 2152 | 0.277 | 0.289 | 0.264 | 0.182 | 0.439 | 0.425 | 0.408 | 0.310 | 0.253 | 0.396 |
| Self-esteem (Good Focal) | 2127 | 0.988 | 1.010 | 0.987 | 0.783 | 1.097 | 1.045 | 1.033 | 0.861 | 0.942 | 1.009 |
| **INTERNAL CONSISTENCY** | | | | | | | | | | | |
| Alpha (Bad Focal) | 2004 | 0.384 | 0.436 | 0.420 | 0.355 | 0.394 | 0.423 | 0.408 | 0.369 | 0.399 | 0.399 |
| Alpha (Good Focal) | 2014 | 0.431 | 0.468 | 0.453 | 0.355 | 0.432 | 0.462 | 0.449 | 0.370 | 0.427 | 0.428 |
| **RELATIONS WITH OTHER IMPLICIT MEASURES** | | | | | | | | | | | |
| *BAD FOCAL* |  |  |  |  |  |  |  |  |  |  |  |
| IAT | 291 | 0.095 | 0.105 | 0.105 | 0.092 | 0.132 | 0.132 | 0.131 | 0.110 | 0.099 | 0.126 |
| GNAT | 295 | 0.084 | 0.069 | 0.083 | 0.103 | 0.077 | 0.073 | 0.081 | 0.092 | 0.085 | 0.081 |
| ST-IAT | 300 | 0.082 | 0.064 | 0.063 | 0.069 | 0.089 | 0.086 | 0.054 | 0.070 | 0.070 | 0.075 |
| SPF | 286 | 0.025 | 0.055 | 0.034 | -0.055 | 0.074 | 0.074 | 0.057 | 0.019 | 0.015 | 0.056 |
| EPT | 275 | 0.053 | 0.010 | 0.037 | 0.065 | 0.040 | 0.018 | 0.038 | 0.059 | 0.041 | 0.039 |
| AMP | 340 | 0.061 | 0.083 | 0.073 | 0.067 | 0.057 | 0.075 | 0.065 | 0.057 | 0.071 | 0.064 |
| SPD | 401 | -0.034 | -0.033 | -0.014 | 0.009 | -0.042 | -0.037 | -0.013 | 0.023 | -0.018 | -0.017 |
|  |  |  |  |  |  |  |  |  |  |  |  |
| *GOOD FOCAL* |  |  |  |  |  |  |  |  |  |  |  |
| IAT | 291 | 0.069 | 0.120 | 0.077 | 0.013 | 0.089 | 0.132 | 0.092 | 0.028 | 0.070 | 0.085 |
| GNAT | 295 | 0.030 | 0.083 | 0.047 | -0.003 | 0.093 | 0.090 | 0.059 | 0.016 | 0.039 | 0.065 |
| ST-IAT | 300 | -0.046 | -0.011 | -0.033 | -0.065 | 0.002 | 0.011 | 0.004 | -0.015 | -0.039 | 0.001 |
| SPF | 286 | 0.002 | 0.037 | -0.009 | -0.054 | 0.001 | 0.040 | 0.004 | -0.039 | -0.006 | 0.002 |
| EPT | 275 | 0.113 | 0.155 | 0.158 | 0.127 | 0.149 | 0.167 | 0.174 | 0.167 | 0.138 | 0.164 |
| AMP | 340 | 0.022 | 0.003 | 0.004 | 0.011 | 0.019 | 0.016 | 0.02 | 0.016 | 0.010 | 0.018 |
| SPD | 401 | -0.029 | -0.037 | -0.032 | -0.021 | -0.001 | -0.014 | -0.006 | 0.001 | -0.030 | -0.005 |
|  |  |  |  |  |  |  |  |  |  |  |  |
| Bad focal average |  | 0.052 | 0.051 | 0.054 | 0.050 | 0.061 | 0.060 | 0.059 | 0.061 | 0.052 | 0.060 |
| Good focal average |  | 0.023 | 0.050 | 0.030 | 0.001 | 0.050 | 0.063 | 0.050 | 0.025 | 0.026 | 0.047 |
| **RELATIONS WITH SELF-REPORT MEASURES AND CRITERION VARIABLES** | | | | | | | | | | | |
| *BAD FOCAL* |  |  |  |  |  |  |  |  |  |  |  |
| Self-Other Preference | 477 | 0.121 | 0.138 | 0.127 | 0.101 | 0.140 | 0.134 | 0.121 | 0.102 | 0.122 | 0.124 |
| Warmth for Self | 479 | 0.012 | 0.051 | 0.031 | -0.002 | 0.034 | 0.042 | 0.024 | 0.002 | 0.023 | 0.026 |
| Warmth for Others | 480 | 0.064 | 0.050 | 0.045 | 0.043 | 0.056 | 0.068 | 0.058 | 0.047 | 0.051 | 0.057 |
| Self-Attributes Questionnaire | 427 | -0.020 | 0.001 | -0.024 | -0.048 | -0.026 | 0.010 | -0.011 | -0.037 | -0.023 | -0.016 |
| Rosenberg Self-Esteem | 472 | 0.133 | 0.172 | 0.174 | 0.156 | 0.166 | 0.176 | 0.182 | 0.173 | 0.159 | 0.174 |
| Recency of Positive Feedback | 479 | 0.034 | 0.015 | 0.030 | 0.042 | 0.028 | 0.015 | 0.027 | 0.035 | 0.030 | 0.026 |
| Recency of Negative Feedback | 479 | 0.032 | 0.024 | 0.003 | -0.012 | 0.016 | 0.019 | -0.002 | -0.029 | 0.012 | 0.001 |
|  |  |  |  |  |  |  |  |  |  |  |  |
| *GOOD FOCAL* |  |  |  |  |  |  |  |  |  |  |  |
| Self-Other Preference | 477 | 0.044 | 0.085 | 0.052 | 0.004 | 0.076 | 0.090 | 0.059 | 0.018 | 0.046 | 0.061 |
| Warmth for Self | 479 | 0.042 | 0.037 | 0.035 | 0.016 | 0.066 | 0.052 | 0.051 | 0.035 | 0.033 | 0.051 |
| Warmth for Others | 480 | 0.069 | 0.094 | 0.086 | 0.069 | 0.077 | 0.084 | 0.076 | 0.061 | 0.080 | 0.075 |
| Self-Attributes Questionnaire | 427 | 0.055 | 0.085 | 0.090 | 0.083 | 0.044 | 0.085 | 0.081 | 0.057 | 0.078 | 0.067 |
| Rosenberg Self-Esteem | 472 | 0.026 | 0.038 | 0.036 | 0.006 | 0.058 | 0.051 | 0.052 | 0.026 | 0.027 | 0.047 |
| Recency of Positive Feedback | 479 | 0.078 | 0.097 | 0.093 | 0.070 | 0.093 | 0.089 | 0.083 | 0.065 | 0.085 | 0.083 |
| Recency of Negative Feedback | 479 | 0.080 | 0.039 | 0.050 | 0.057 | 0.055 | 0.040 | 0.046 | 0.047 | 0.057 | 0.047 |
|  |  |  |  |  |  |  |  |  |  |  |  |
| Bad focal average |  | 0.047 | 0.057 | 0.049 | 0.035 | 0.052 | 0.058 | 0.050 | 0.037 | 0.047 | 0.049 |
| Good focal average |  | 0.049 | 0.059 | 0.055 | 0.038 | 0.059 | 0.061 | 0.056 | 0.039 | 0.051 | 0.054 |
| **RELATIONS WITH EXTRANEOUS INFLUENCE** | | | | | | | | | | | |
| *BAD FOCAL* |  |  |  |  |  |  |  |  |  | average of absolute values | |
| Relation with average reciprocal | 2048 | -0.030 | 0.099 | -0.140 | -0.338 | -0.009 | 0.119 | -0.115 | -0.320 | -0.102 | -0.081 |
| Relation with average log | 2048 | 0.036 | -0.095 | 0.190 | 0.445 | -0.011 | -0.133 | 0.151 | 0.425 | 0.144 | 0.108 |
| Relation with average latency | 2048 | 0.038 | -0.069 | 0.213 | 0.487 | -0.010 | -0.109 | 0.174 | 0.472 | 0.167 | 0.132 |
|  |  |  |  |  |  |  |  |  |  |  |  |
| *GOOD FOCAL* |  |  |  |  |  |  |  |  |  | average of absolute values | |
| Relation with average reciprocal | 2066 | 0.053 | 0.090 | -0.124 | -0.318 | 0.029 | 0.071 | -0.131 | -0.316 | -0.075 | -0.087 |
| Relation with average log | 2066 | -0.067 | -0.089 | 0.161 | 0.398 | -0.052 | -0.081 | 0.165 | 0.402 | 0.101 | 0.109 |
| Relation with average latency | 2066 | -0.083 | -0.079 | 0.165 | 0.420 | -0.070 | -0.073 | 0.167 | 0.420 | 0.106 | 0.111 |
