# Supplementary material for: Understanding and Using the Brief Implicit Association Test: Recommended Scoring Procedures
Source: PLoS One. 2014 Dec 8;9(12):e110938. doi: 10.1371/journal.pone.0110938 (PMC4259300; doi:10.1371/journal.pone.0110938)
Supplement: S3 Table — Comparison of fast and slow latency treatments across evaluation criteria for race. Magnitude of main effect is Cohen's d of average BIAT score, others are correlation coefficients. Correlations averaged after Fisher's z-transformation and then converted back to a correlation. (DOCX) [file pone.0110938.s004.docx]

Table S3. Comparison of fast and slow latency treatments across evaluation criteria for race. Magnitude of main effect is Cohen's d of average BIAT score, others are correlation coefficients. Correlations averaged after Fisher's z-transformation and then converted back to a correlation.

|  | Fast Latency Treatment | | | | | | | Slow Latency Treatment | | |
| --- | --- | --- | --- | --- | --- | --- | --- | --- | --- | --- |
|  | Deleting | | | Recoding | Deleting | | | Recoding | | |
|  | D400 | D200 | D none | D400 | D400 + D2000 | D400 + D3000 | D400 + D4000 | D400 + D2000 | D400 + D3000 | D400 + D4000 |
| **MAGNITUDE OF MAIN EFFECT** | 0.450 | 0.444 | 0.444 | 0.445 | 0.460 | 0.451 | 0.453 | 0.454 | 0.447 | 0.446 |
| **KNOWN GROUP DIFFERENCE** (Race) | 0.184 | 0.184 | 0.183 | 0.183 | 0.173 | 0.183 | 0.186 | 0.183 | 0.187 | 0.185 |
| **KNOWN GROUP DIFFERENCE** (Political ID) | 0.199 | 0.204 | 0.206 | 0.205 | 0.207 | 0.208 | 0.201 | 0.213 | 0.208 | 0.206 |
| **INTERNAL CONSISTENCY** (alpha) | 0.569 | 0.575 | 0.576 | 0.575 | 0.566 | 0.564 | 0.568 | 0.578 | 0.578 | 0.577 |
|  |  |  |  |  |  |  |  |  |  |  |
| **RELATIONS WITH OTHER IMPLICIT MEASURES** | | | | | | | | | | |
| IAT | 0.404 | 0.415 | 0.412 | 0.415 | 0.441 | 0.430 | 0.421 | 0.436 | 0.423 | 0.418 |
| GNAT | 0.358 | 0.360 | 0.360 | 0.360 | 0.350 | 0.360 | 0.360 | 0.357 | 0.362 | 0.361 |
| ST-IAT | 0.375 | 0.379 | 0.379 | 0.379 | 0.401 | 0.390 | 0.383 | 0.389 | 0.383 | 0.379 |
| SPF | 0.312 | 0.318 | 0.318 | 0.318 | 0.301 | 0.306 | 0.311 | 0.311 | 0.316 | 0.318 |
| EPT | 0.308 | 0.324 | 0.323 | 0.322 | 0.313 | 0.315 | 0.307 | 0.321 | 0.321 | 0.321 |
| AMP | 0.227 | 0.227 | 0.227 | 0.227 | 0.219 | 0.229 | 0.227 | 0.226 | 0.228 | 0.228 |
| SPD | 0.297 | 0.297 | 0.296 | 0.297 | 0.310 | 0.311 | 0.304 | 0.309 | 0.300 | 0.298 |
|  |  |  |  |  |  |  |  |  |  |  |
| Average | 0.327 | 0.333 | 0.332 | 0.332 | 0.335 | 0.336 | 0.332 | 0.337 | 0.335 | 0.333 |
|  |  |  |  |  |  |  |  |  |  |  |
| **RELATIONS WITH SELF-REPORT MEASURES AND CRITERION VARIABLES** | | | | | | | | | | |
| Black-White Preference | 0.278 | 0.278 | 0.277 | 0.278 | 0.269 | 0.274 | 0.275 | 0.281 | 0.280 | 0.278 |
| Warmth for Blacks | 0.115 | 0.116 | 0.115 | 0.116 | 0.103 | 0.104 | 0.108 | 0.110 | 0.113 | 0.116 |
| Warmth for Whites | 0.163 | 0.164 | 0.164 | 0.163 | 0.157 | 0.162 | 0.164 | 0.166 | 0.164 | 0.163 |
| Avg liking of 5 Black people | 0.133 | 0.143 | 0.143 | 0.142 | 0.143 | 0.135 | 0.132 | 0.148 | 0.142 | 0.142 |
| Avg liking of 5 White people | 0.206 | 0.211 | 0.211 | 0.210 | 0.222 | 0.190 | 0.185 | 0.208 | 0.205 | 0.209 |
| Modern Racism Scale | 0.334 | 0.335 | 0.335 | 0.336 | 0.336 | 0.330 | 0.336 | 0.338 | 0.337 | 0.337 |
| Contact with Black people | 0.111 | 0.107 | 0.106 | 0.108 | 0.104 | 0.099 | 0.102 | 0.103 | 0.106 | 0.107 |
| Right-Wing Authoritarianism | 0.237 | 0.248 | 0.250 | 0.250 | 0.236 | 0.243 | 0.237 | 0.260 | 0.255 | 0.253 |
|  |  |  |  |  |  |  |  |  |  |  |
| Average | 0.198 | 0.202 | 0.201 | 0.202 | 0.198 | 0.193 | 0.194 | 0.203 | 0.202 | 0.202 |
|  |  |  |  |  |  |  |  |  |  |  |
| **RELATIONS WITH EXTRANEOUS INFLUENCE** | | | | | | | | | | |
| Relation with average reciprocal | 0.019 | 0.012 | 0.012 | 0.013 | -0.006 | -0.004 | 0.009 | -0.023 | -0.008 | 0.004 |
| Relation with average log | -0.018 | -0.011 | -0.010 | -0.011 | 0.015 | 0.011 | -0.006 | 0.032 | 0.016 | -0.0001 |
| Relation with average latency | -0.025 | -0.019 | -0.019 | -0.020 | 0.014 | 0.010 | -0.010 | 0.027 | 0.012 | -0.006 |
