# Supplementary material for: Understanding and Using the Brief Implicit Association Test: Recommended Scoring Procedures
Source: PLoS One. 2014 Dec 8;9(12):e110938. doi: 10.1371/journal.pone.0110938 (PMC4259300; doi:10.1371/journal.pone.0110938)
Supplement: S4 Table — Comparison of fast and slow latency treatments across evaluation criteria for self-esteem. Magnitude of main effect is Cohen's d of average BIAT score, others are correlation coefficients. Correlations averaged after Fisher's z-transformation and then converted back to a correlation. (DOCX) [file pone.0110938.s005.docx]

Table S4. Comparison of fast and slow latency treatments across evaluation criteria for self-esteem. Magnitude of main effect is Cohen's d of average BIAT score, others are correlation coefficients. Correlations averaged after Fisher's z-transformation and then converted back to a correlation.

|  | Fast Latency Treatment | | | | | | | Slow Latency Treatment | | |
| --- | --- | --- | --- | --- | --- | --- | --- | --- | --- | --- |
|  | Deleting | | | Recoding | Deleting | | | Recoding | | |
|  | D400 | D200 | D none | D400 | D400 + D2000 | D400 + D3000 | D400 + D4000 | D400 + D2000 | D400 + D3000 | D400 + D4000 |
| **MAGNITUDE OF MAIN EFFECT** | 1.164 | 1.163 | 1.160 | 1.162 | 1.178 | 1.186 | 1.167 | 1.190 | 1.173 | 1.166 |
| **INTERNAL CONSISTENCY** (alpha) | 0.432 | 0.429 | 0.428 | 0.429 | 0.444 | 0.433 | 0.431 | 0.474 | 0.456 | 0.461 |
|  |  |  |  |  |  |  |  |  |  |  |
| **RELATIONS WITH OTHER IMPLICIT MEASURES** | | | | | | | | | | |
| IAT | 0.089 | 0.094 | 0.094 | 0.093 | 0.155 | 0.121 | 0.102 | 0.110 | 0.098 | 0.096 |
| GNAT | 0.093 | 0.094 | 0.094 | 0.093 | 0.114 | 0.099 | 0.096 | 0.092 | 0.093 | 0.093 |
| ST-IAT | 0.002 | 0.001 | 0.011 | 0.009 | 0.045 | 0.016 | 0.011 | 0.021 | 0.012 | 0.009 |
| SPF | 0.001 | 0.001 | 0.001 | 0.002 | 0.035 | 0.019 | -0.003 | 0.010 | 0.004 | 0.003 |
| EPT | 0.149 | 0.144 | 0.143 | 0.146 | 0.159 | 0.145 | 0.147 | 0.151 | 0.148 | 0.147 |
| AMP | 0.019 | 0.027 | 0.027 | 0.026 | 0.017 | 0.018 | 0.015 | 0.027 | 0.028 | 0.029 |
| SPD | -0.001 | 0.001 | -0.0003 | -0.0003 | 0.016 | 0.003 | 0.002 | 0.003 | -0.003 | -0.003 |
|  |  |  |  |  |  |  |  |  |  |  |
| Average | 0.050 | 0.052 | 0.053 | 0.053 | 0.078 | 0.060 | 0.053 | 0.059 | 0.054 | 0.054 |
|  |  |  |  |  |  |  |  |  |  |  |
| **RELATIONS WITH SELF-REPORT MEASURES AND CRITERION VARIABLES** | | | | | | | | | | |
| Self-Other Preference | 0.076 | 0.080 | 0.081 | 0.080 | 0.062 | 0.064 | 0.067 | 0.068 | 0.072 | 0.074 |
| Warmth for Self | 0.066 | 0.069 | 0.070 | 0.069 | 0.061 | 0.058 | 0.063 | 0.062 | 0.064 | 0.065 |
| Warmth for Others | 0.077 | 0.079 | 0.079 | 0.079 | 0.081 | 0.073 | 0.074 | 0.077 | 0.078 | 0.079 |
| Self-Attributes Questionnaire | 0.044 | 0.048 | 0.047 | 0.047 | 0.085 | 0.073 | 0.063 | 0.068 | 0.056 | 0.049 |
| Rosenberg Self-Esteem | 0.058 | 0.061 | 0.057 | 0.058 | 0.085 | 0.086 | 0.076 | 0.075 | 0.067 | 0.062 |
| Recency of Positive Feedback | 0.093 | 0.091 | 0.091 | 0.091 | 0.116 | 0.092 | 0.084 | 0.093 | 0.089 | 0.091 |
| Recency of Negative Feedback | 0.055 | 0.051 | 0.050 | 0.051 | 0.025 | 0.053 | 0.054 | 0.044 | 0.049 | 0.051 |
|  |  |  |  |  |  |  |  |  |  |  |
|  |  |  |  |  |  |  |  |  |  |  |
| Average | 0.059 | 0.060 | 0.059 | 0.059 | 0.064 | 0.062 | 0.060 | 0.061 | 0.059 | 0.059 |
|  |  |  |  |  |  |  |  |  |  |  |
| **RELATIONS WITH EXTRANEOUS INFLUENCE** | | | | | | | | | | |
| Relation with average reciprocal | 0.029 | 0.034 | 0.030 | 0.030 | -0.043 | -0.005 | 0.009 | -0.028 | 0.002 | 0.016 |
| Relation with average log | -0.052 | -0.057 | -0.057 | -0.056 | 0.024 | -0.013 | -0.024 | 0.015 | -0.018 | -0.036 |
| Relation with average latency | -0.07 | -0.074 | -0.074 | -0.073 | 0.012 | -0.024 | -0.036 | 0.003 | -0.030 | -0.05 |
